# Nasal septum deviation after orthognathic Le Fort I osteotomy: a systematic review and meta-analysis

**DOI:** 10.1186/s40902-025-00483-8

**Published:** 2025-10-09

**Authors:** Amir-Ali Yousefi-Koma, Anahita Moscowchi, Mahdi Kadkhodazadeh, Reza Tabrizi

**Affiliations:** 1 Dentofacial Deformities Research Center, Research Institute of Dental Sciences, Shahid Beheshti University of Medical Sciences, Tehran, Islamic Republic of Iran; 2 Department of Oral and Maxillofacial Surgery, Shahid Beheshti University of Medical Sciences, Tehran, Islamic Republic of Iran; 3 Dental Research Center, Research Institute of Dental Sciences, Shahid Beheshti University of Medical Sciences, Tehran, Islamic Republic of Iran; 4 Department of Periodontics, Shahid Beheshti University of Medical Sciences, Tehran, Islamic Republic of Iran

**Keywords:** Osteotomy, Le Fort [MeSH], Orthognathic Surgery [MeSH], Oral Surgery, Maxillofacial Plastic Surgeries, Nasal Septum [MeSH], Nose [MeSH], Septal Deviation, Alar Base

## Abstract

**Background:**

Orthognathic Le Fort I osteotomy (LF-IO) reinstates an accurate anatomical and functional interrelation between the facial skeletal structures. There are numerous reports in the literature regarding nasal ventilation after LF-IO, yet the number of studies focused on nasal septum angle/deviation is limited.

**Purpose:**

This systematic review was designed to gather and analyze all of the human studies that have investigated nasal septum angle and deviation before and after LF-IO.

**Data sources:**

An electronic search was executed in Medline via PubMed, Web of Science, Scopus, and Google Scholar to identify eligible studies Only in English language up to July 10, 2025.

**Study selection:**

Randomized and non-randomized human clinical studies on adult patients undergoing single-piece or segmental LF-IO with no history of facial traumas and/or anomalies.

**Data extraction and synthesis:**

Random-effects model analysis was used in all cases. The risk of publication bias was assessed using a funnel plot and Egger’s test. All statistical analyses were executed using Comprehensive Meta-analysis software with the significance threshold of 0.05.

**Main outcomes and measures:**

Changes in nasal septum angle measured in degrees and through radiography and alar base width changes measured in millimeters.

**Results:**

One non-randomized clinical trial, ten retrospective and One prospective cohort studies were included; 579 patients were enrolled with a gender ratio of 217:362 (male:female) and an age range of 16 to 56 years old. Four of the included studies had high and eight had moderate qualities regarding their risk of bias. Most patients underwent LF-IO to correct Class III malocclusions. Single-piece LF-IO combined with alar base cinch suture was the most popular surgical procedure. Frontal sections in computed tomography before and 12 months after LF-IO was the most utilized evaluation method. Six studies were selected for various meta-analyses with significantly low publication bias. Releasing nasal septum during LF-IO leads to significant increases in septum angle.

**Conclusions and relevance:**

LF-IO, especially maxillary advancement, significantly increases nasal septum angle and alar base width. A clear definition and diagnosis protocol must be established for nasal septum deviation. Future studies must focus on highlighting a fine line between significant and insignificant changes in nasal septum after LF-IO.

**Supplementary Information:**

The online version contains supplementary material available at 10.1186/s40902-025-00483-8.

## Introduction

Orthognathic surgeries are utilized to reinstate an accurate anatomical and functional interrelation between the facial skeletal structures affected by anatomical malpositions, anomalies, and/or traumas [1–3]. Orthognathic surgeries combined with orthodontic treatments restore the loss of function and esthetics in the maxilla, the mandible and in some cases even in the nose, chin, and cheekbones [4–6]. Wassmund recognized the necessity of correcting midface malpositions through total maxillary osteotomy; Le Fort I osteotomy (LF-IO) corrects maxillary position in three dimensions and was first executed by Wassmund in 1921 with the aim of counterbalancing dentofacial anomalies [7–9]. The Le Fort I plane extends posteriorly onward the tooth roots and involves the pterygomaxillary connection [9]. The LF-IO technique allows for the displacement of maxilla in sagittal, vertical and transverse directions, consequently, enabling the treatment of skeletal class II and III dysgnathia, maxillary excess, and hypoplasia [10]. During LF-IO, first, the nasal septum gets detached from the maxilla, then the down-fracture, displacement, and osteo-synthetic fixation/correction of the maxilla in its new position gets executed, and finally, the nasal septum will be refixed to the anterior nasal spine [11].

**Fig. 1:**
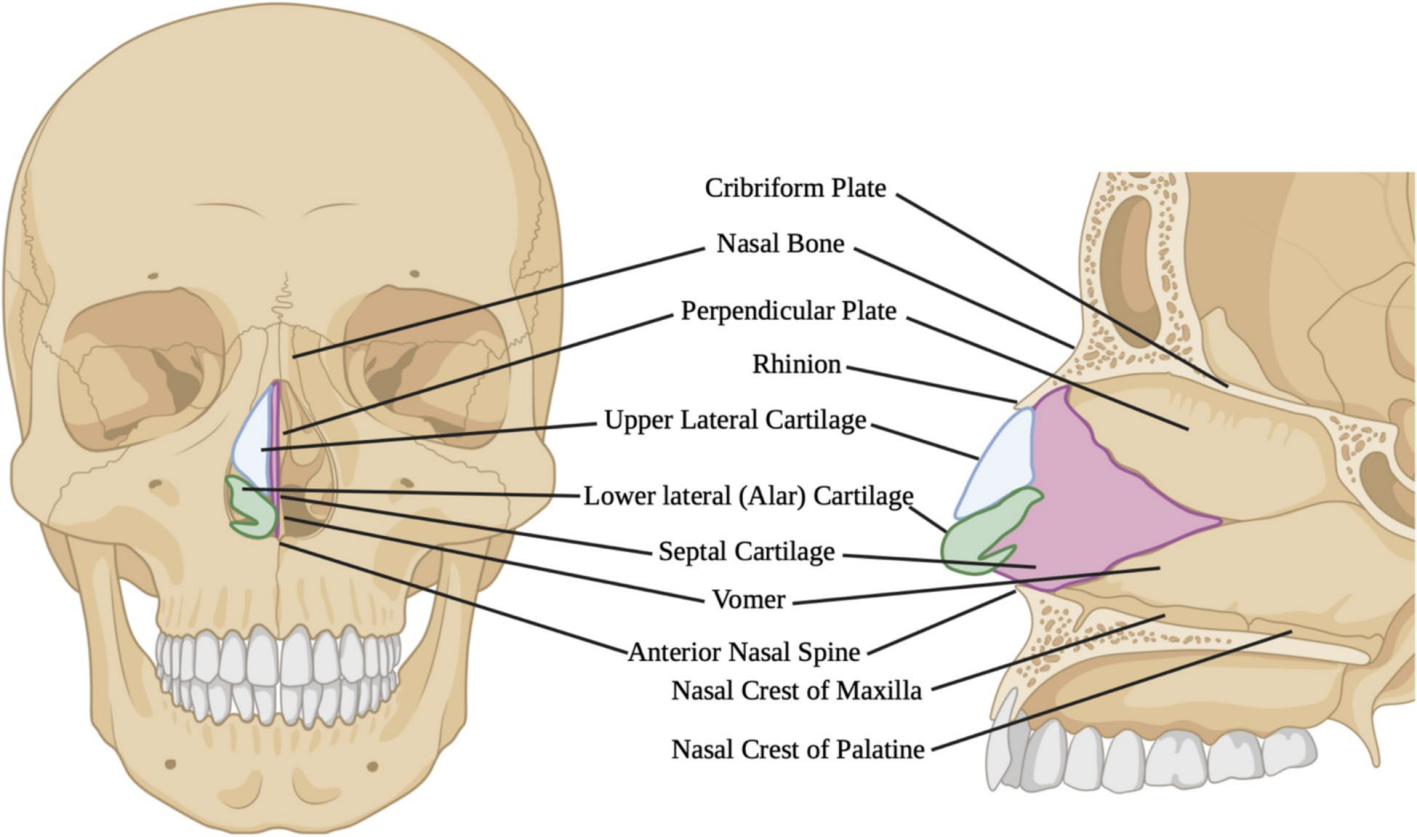
Nasal septum anatomy (Illustration drawn by the authors of this paper, using the BioRender online tools at www.biorender.com)

The nasal septum is a crucial part of what constitutes the overall form of the nose [12, 13]. Due to the highly intertwined nature of the nasal shape/form, function, and its complicated interrelation with the maxilla, it is crucial for clinicians and researchers that focus on orthognathic surgeries to have a strong comprehension of the nasal internal and external anatomy, specifically the nasal septum. The nasal septum is consisted of two portions; bony and cartilaginous. The bony nasal septum is formed in three sections (Fig. 1): (1) the upper third is formed by the perpendicular plate of the ethmoid bone, which articulates anteriorly accompanied by an inward spine from the nasal bones, and continues superiorly alongside the frontal bone and cribriform plate; (2) the middle third is shaped by the perpendicular plate articulating inferiorly with the vomer bone and the quadrilateral septal cartilage; and (3) the lower third is consisted of the keel-shaped vomer bone extending from maxilla posteriorly through the sphenoid rostrum, and to the nasal crest of the palatine bones anteriorly; premaxillary wings fuse with the vomer in the midline in order to form a groove that acts as a rest for the inferior edge of the quadrilateral cartilage. The cartilaginous septum provides the necessary support for the nasal dorsum directly below the rhinion with a tail caudally extended to the supra-tip sector. The upper lateral cartilages combined with the dorsal cartilaginous septum emerge as a single embryological unit sharing a common peri-chondrial lining. The upper lateral cartilages deviate from the septum in order to form a fibrous aponeurosis (Fig. 1) [14, 15]. The nasal septum divides the nasal airway in a symmetrical manner, while defining the height and position of the apex of the nose. Due to the extremely close relation/connection between the maxilla and the nose, LF-IO can easily affect both the bony and the cartilaginous segments of the nose [16]. Surgical maxillary movements in different directions may cause alar base widening, nasal obstruction, an increase in nasolabial angle, nasal septum deviation, and perforation [17]. Nasal septum dictates the height and position of the tip of the nose, providing nasal ventilation. Deficient reduction of maxillary bony crest and/or nasal cartilage throughout LF-IO may lead to the displacement of nasal septum from midline. Nasal septum deviation can cause restricted nasal breathing and consequently, significantly diminish patients’ overall quality of life; in some cases, subsequent surgical amendment of the nasal septum might be necessary after LF-IO [18–20].

There are numerous reports in the literature regarding the nasal form and nasal ventilation after LF-IO, yet the number of studies focused on investigating the nasal septum angle and deviation post LF-IO is relatively limited [21]. Even though clinicians perform LF-IO on a regular basis, there is still no clear answer/guideline to predict the direct and indirect effects of LF-IO on nasal septum angle/deviation and other nasal septum complications. The identification of such a crucial risk factor, nasal septum deviation, will definitely help in the treatment planning for orthognathic surgery candidates. To the best of the authors’ knowledge, there are no review articles of any kind (e.g., systematic reviews, scoping reviews, narrative reviews, reviews of the literature) in the literature that have investigated the adverse effects of LF-IO on the nasal anatomy and function with a focus on nasal septum angle and deviation post-surgery. This systematic review was designed and executed to gather all of the human studies, with reliable study designs and sample sizes, that have investigated nasal septum angle and deviation before and after LF-IO.

## Materials and methods

This study has been prepared and organized according to the preferred reporting items for systematic reviews and meta-analyses (PRISMA) 2020 guidelines [22]. This systematic review has been registered at PROSPERO (Registration ID: CRD42024618159). The study question according to the PICO format was as follows: comparing the state of nasal septum angle/deviation (C and O) before and after the LF-IO procedure (I) in adult human patients (P).

### Eligibility criteria

#### Types of studies

Randomized and non-randomized clinical trials, retrospective, and prospective cohort studies.

#### Population

Adult human patients eligible for orthognathic surgeries (i.e., 16 years old and above).

#### Intervention

Single-piece or segmental LF-IO with or without other orthognathic surgeries (e.g., sagittal split ramus osteotomy (SSRO), rapid surgical maxillary expansion) performed for either class II or class III malocclusions (i.e., anterior or non-anterior movements of the maxilla).

#### Types of outcome measures

The primary outcome as the main focus of this review was the measurement of nasal septum angle and deviation before and after LF-IO through radiographic evaluations (e.g., computed tomography (CT), cone beam CT (CBCT), lateral cephalometry, anterior–posterior cephalometry). Moreover, the changes of alar base width after LF-IO were considered the secondary outcomes investigated in this systematic review; ideally, these changes must have been reported in actual numbers in millimeters compared to the pre-LF-IO measurements through soft tissue evaluations using the same reference points.

### Information sources and search strategy

An electronic search was executed in Medline via PubMed, Web of Science, Scopus, and Google Scholar to identify eligible studies Only in English language. The search was included of articles up to July 10, 2024. Search queries displayed in Table 1 were considered for electronic search.

**Table 1:** Search queries

*Data base*	*Date*	*Search query*	*Results*
Medline via PubMed	July 10, 2025	(“Orthognathic Surgical Procedures” [MeSH] or “Orthognathic Surgery” [MeSH] or “Surgery, Oral” [MeSH] or “Oral Surgical Procedures” [MeSH] or “Osteotomy, Le Fort” [MeSH] or “maxillary reposition” or “maxilla reposition” or “Le Fort” or “Le Fort I”) AND (“Nose Deformities, Acquired” [MeSH] or “Nasal Septum” [MeSH] or “Nasal Septal Deviation” or “Nasal Septum Deviation”)	219
Scopus	July 10, 2025	TITLE-ABS-KEY ((Lefort I osteotomy) OR (Lefort) OR (Lefort I) OR (Orthognathic surgery)) AND ((Nasal septum) OR (Septal deviation))	102
Web of Science	July 10, 2025	(ALL = (Le Fort I)) AND (ALL = (Nasal septum deviation) OR ALL = (nasal septal deviation) OR ALL = (nasal septum angle) OR ALL = (nasal septal angle))	28
Google Scholar	July 10, 2025	“Le Fort I” AND “Nasal Septum”	415

### Study selection and data collection

Two reviewers (AY and AM) independently screened the titles and abstracts of articles and included/excluded articles based on the mentioned exclusion criteria. Selected articles were then fully read to see if they passed our inclusion criteria. In case of any disagreements, a third reviewer (RT) was consulted. The demographic and methodological details along with the outcomes from selected studies were then extracted and tabulated. In case of any conflicts, a third expert (RT) was consulted to find common ground.

### Data items

The collected items were as follows: (1) authors’ name; (2) year of publishment; (3) type of study; (4) number of patients; (5) patients’ gender ratio; (6) patients’ mean age and/or age range; (7) study groups; (8) surgery rationale (e.g., class II malocclusion, class III malocclusion, rapid maxillary expansion); (9) exclusion criteria specific to each study (e.g., congenital deformities, traumatic deformities, history of septoplasty); (10) surgical intervention (i.e., single-piece LF-IO or segmental LF-IO); (11) other orthognathic surgeries in combination with LF-IO (e.g., SSRO); (12) cartilage manipulation; (13) anesthesia methodologies; (14) post-surgical skeletal fixation methodologies; (15) study variables; (16) methods of assessment; (17) evaluation periods; (18) results and conclusions.

### Synthesis methods

The rate of septum deviation was determined by analyzing studies that documented the incidence of outcome. Standardized mean differences and 95% confidence intervals (CIs) were used to report changes in septal angle and alar base width. Odds ratio was also reported for the changes in alar base width. In cases of sufficient details about the surgical procedure and evaluation methods, subgroup analyses were also performed.

Given that multiple factors can affect the incidence of septal deviation and angle changes, random-effects model analysis was used in all cases. The risk of publication bias was assessed using a funnel plot and Egger’s test (the significance level was set at 0.10). All statistical analyses were executed using Comprehensive Meta-analysis software (Version 3, Biostat Inc., NJ, USA) with the significance threshold of 0.05.

### Quality assessment

The methodological index for non-randomized studies (MINORS) was utilized to assess the risk of bias and quality for all of the included studies [23]. Two reviewers (AY and AM) independently evaluated each study using the prefabricated questions of the mentioned quality assessment tool. In case of any heterogeneities in the results, a third expert (RT) was consulted.

## Results

### Study selection

**Fig. 2:**
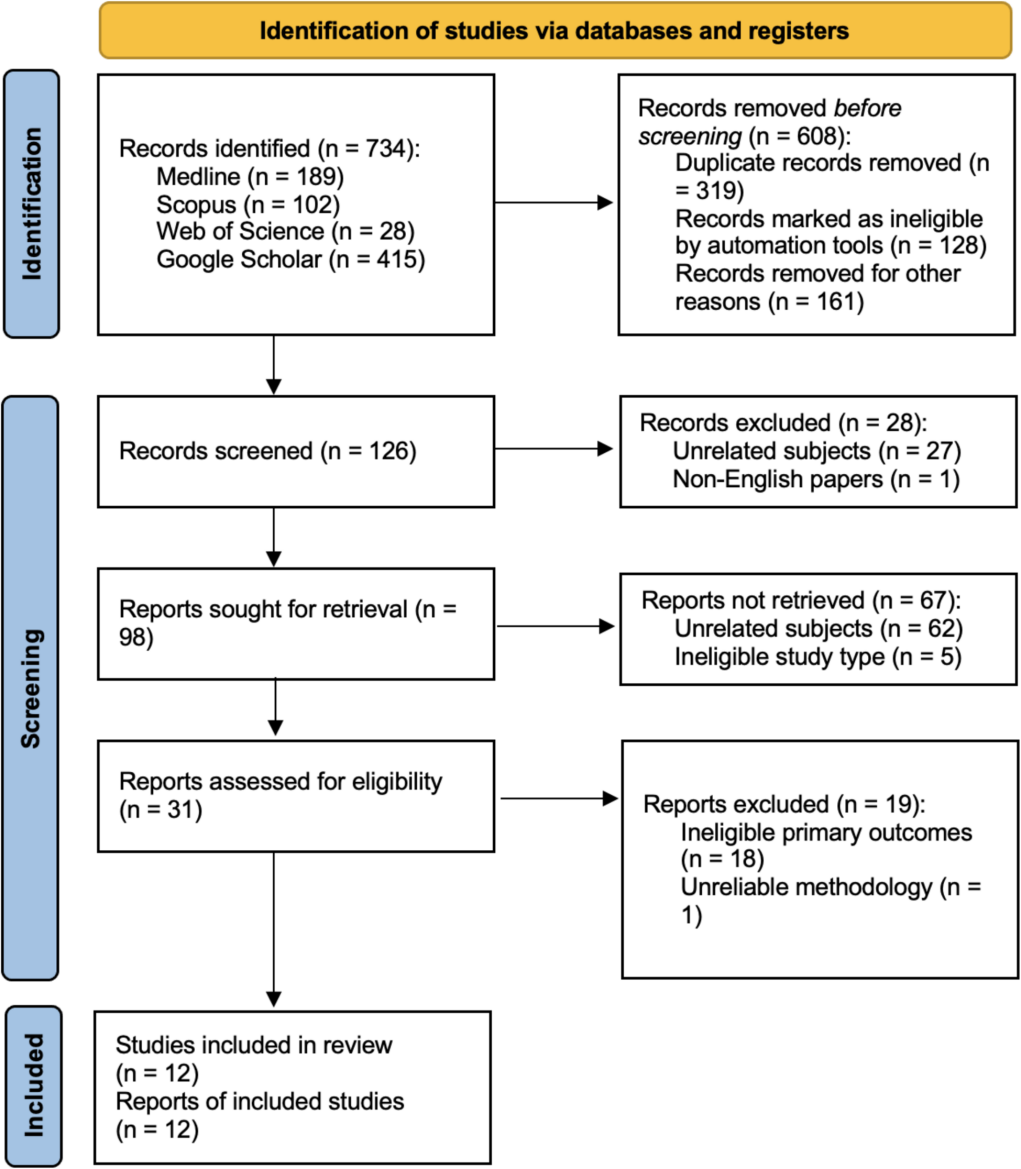
The PRISMA 2020 flow diagram

Database screening was conducted and a total of 734 papers were initially identified (Fig. 2). After the removal of non-English papers, duplicates, ineligible study types, and unrelated subjects, a total of 31 papers were screened for eligibility. Eighteen papers were excluded due to not investigating the primary outcomes of nasal septum angle/deviation in any form. One study was excluded due to having an extremely unreliable study design and methodology in regard to the evaluation of nasal septum angle/deviation. A total of 1 non-randomized clinical trial, 10 retrospective cohort and 1 prospective cohort studies were included in this review (Fig. 2). There were no randomized clinical trials in the literature in accordance with the eligibility criteria of this review. The 12 included studies were published between 2016 and 2024; 2016 (*n* = 2) [21, 24], 2017 (*n* = 1) [25], 2020 (*n* = 3) [26–28], 2022 (*n* = 1) [29], 2023 (*n* = 3) [3, 18, 30], and 2024 (*n* = 2) [31, 32]. The included studies came from 6 different countries; Turkey (*n* = 4) [3, 26, 29, 31], Japan (*n* = 3) [21, 28, 30], The Republic of Korea (*n* = 2) [25, 27], India (*n* = 1) [18], Denmark (*n* = 1) [24], and Germany (*n* = 1) [32]. The 12 included studies were published in 9 different journals; The Journal of Craniofacial Surgery (*n* = 4) [25, 27, 29, 30], American Association of Oral and Maxillofacial Surgeons (*n* = 1) [3], Journal of Cranio-maxillo-facial Surgery (*n* = 1) [21], Nigerian Journal of Clinical Practice (*n* = 1) [26], Journal of Maxillofacial and Oral Surgery (*n* = 1) [18], Oral Surgery Oral Medicine Oral Pathology Oral Radiology (*n* = 1) [24], Annals of Plastic Surgery (*n* = 1) [31], Journal of Plastic, Reconstructive and Aesthetic Surgery (*n* = 1) [28], and Medicina (*n* = 1) [32].

### Results of individual studies

The patient demographics and methodological details along with all of the key results and conclusions of all of the twelve included studies are detailed and tabulated in Supplementary Table 1.

### Study characteristics

#### Study design

One of the included studies was a non-randomized clinical trial [32], 10 of them were retrospective cohorts [3, 18, 21, 25–31], and one of them was a prospective cohort study [24] (Supplementary Table 1).

#### Demographics

A total of 579 patients were enrolled in the twelve included studies; the gender ratio of the enrolled patients was 217:362 (male:female). Patients’ age ranged from 16 to 56 years old; however, three studies did not report their patients’ age range [25, 26, 29]. Only one study failed to report their patients’ mean average age [31]. Patients’ mean age in the remaining eleven studies ranged from 20.5 [24] to 30.32 [18] years old (Supplementary Table 1).

#### Patient enrollment exclusion criteria

Two of the included studies did not specify their exclusion criteria [21, 25]. In the remaining ten studies, the following exclusion criteria were reported: [1] history of septoplasty, rhinoplasty, or turbinoplasty (*n* = 9) [3, 18, 24, 26–31]; (2) congenital craniofacial deformities (e.g., cleft lip, palate, and jaw) (*n* = 7) [3, 26, 28–32]; (3) post-traumatic deformities (*n* = 7) [3, 18, 26, 27, 29–31]; (4) craniofacial syndromes/anomaly (*n* = 6) [18, 24, 26, 27, 30, 32]; (5) previous orthognathic surgeries (*n* = 6) [24, 26, 29–32]; (6) type 4 (S-shaped) septum deviation (*n* = 1) [31].

#### Surgical rationale and procedure

Out of the twelve included studies, three of them did not specify their surgical rationale [18, 29, 30]. In the remaining nine studies, six different surgical rationale were reported: (1) skeletal class III (*n* = 6) [3, 24, 26, 28, 31, 32]; (2) skeletal class II (*n* = 2) [3, 32]; (3) maxillary vertical excess/deficiency (*n* = 3) [3, 25, 27]; (4) open bite (*n* = 2) [21, 32]; (5) bi-maxillary asymmetry (*n* = 1) [21]; (6) maxillary expansion (*n* = 1) [24]. The anesthesia methodologies were specified in a total of seven studies and all of them used the same nasotracheal technique [3, 24, 26, 29–32]. Only five studies reported the details regarding the manipulation/resection of the cartilaginous septum during the LF-IO procedure; the cartilaginous segment was either reduced/shortened (*n* = 2) [3, 32] or simply separated/resected (*n* = 3) [25, 27, 30]. All of the data regarding different surgical methodologies are showcased in Table 2. The nasal septum was released in all of the participants in 10 studies [3, 18, 21, 25–27, 29–32], while in the remaining two studies, the nasal septum was not released in all participants; (1) Jensen et al. performed tooth borne and bone borne surgically assisted rapid maxillary expansion through LF-IO and had two study groups: (1.A) with the release of the nasal septum (*n* = 10), and (1.B) without the release of the nasal septum (*n* = 10) [24]; (2) Yamashsita et al. performed LF-IO either the conventional way (*n* = 19) or in a sub-spinal style without manipulating the nasal septum or the anterior nasal spine (ANS) (*n* = 20) [28].

**Table 2:** Surgical procedures

*Number*	*Surgical procedure*	*Number of patients*	*References*
1	Segmental LF-IO	10	[24]
2	Single-piece LF-IO + Alar base suture	236	[3, 18, 21, 25, 29–31]
3	Segmental LF-IO + Alar base suture	7	[18]
4	Single-piece LF-IO + BSSRO + Alar base suture	135	[3, 21, 27]
5	Single-piece LF-IO + BSSRO + Alar base suture + V–Y closure	59	[26, 28]
6	Sub-spinal single-piece LF-IO + BSSRO + Alar base suture + V–Y closure	20	[28]

*Abbreviations*: Le Fort I osteotomy (*LF-IO*), and bilateral sagittal split ramus osteotomy (*BSSRO*)

#### Study variables

A number of skeletal and soft tissue factors regarding the nasal septum angle/deviation and other nasal aspects were evaluated in the included studies pre- and post-operatively. To simplify further evaluations and analyses, all of the study variables that had been evaluated in more than one study are all listed in Table 3 along with their methods of assessment and evaluation periods. It is worth noting that all of the study variables and the analyses executed on them were only worthy of analysis if the included studies reported statistically significant/insignificant differences of the mentioned study variables at a certain evaluation period after the surgery; any kind of subjective evaluation of the shape and function of the nose, reported by either the clinicians or the participants, were not included in this systematic review and meta-analysis.

**Table 3:** Study variables

*Number*	*Study variables*	*Methods of assessment*	*Evaluation periods*	*References*
1	Nasal septum deviation	CT	T0 and T12	[21]
		CBCT	TS and T6	[24]
			T0 and T5	[25]
			T0 and T6	[31, 32]
			T0 and T12	[3, 18, 27]
		P-A and lateral cephalometry	T0 and T3	[26]
2	Nasal septum angle (anterior/middle/posterior)	CT	T0 and T12	[21, 30]
3	Nasal cavity height and width	CT	T0 and T6	[29]
			T0 and T12	[21]
		P-A and lateral cephalometry	T0 and T3	[26]
4	Alar base width	CT	T0 and T6	[29]
		CBCT	T0 and T6	[31]
			T0 and T12	[28]
5	Nasolabial angle	CBCT	T0 and T6	[31]
			T0 and T12	[28]
6	Nasal width	CBCT	T0 and T6	[31]
			T0 and T12	[28]
7	Nasal tip protrusion	CT	T0 and T6	[29]
		CBCT	T0 and T12	[28]

*Abbreviations: *Posterior-anterior cephalometry (*P-A*), right before surgery (*T0*), right after surgery (*TS*), 3 months after surgery (T3), 6 months after surgery (*T6*) and 12 months after surgery (*T12*)

### Reported outcomes

As displayed in Tables 2 and 3, studies were categorized based on both their surgical procedures and their study variables. In each of the following sections, an overall description of the reported outcomes of the included studies will be presented, followed by the meta-analyses of the studies that could have been chosen for further analytical evaluations.

#### Nasal septum angle/deviation

Ten of the included studies had followed similar techniques and methodologies in investigating the nasal septum angle and deviation before and after LF-IO [3, 18, 21, 24–27, 30–32] (Tables 2 and 3). Six of the mentioned studies reported significant changes in nasal septum angle after LF-IO; 4 of these studies evaluated and compared the nasal septum angle right before surgery and at 12 months post-surgery [3, 18, 27, 30], while two of them compared the nasal septum angle at 6 months post-surgery versus right before surgery [31, 32]. The other four studies that evaluated nasal septum angle/deviation and reported insignificant changes in nasal septum angle after surgery all had different evaluation periods at 3 months [26], 5 months [24, 25], 6 months [24], and 12 months [21] post-surgery. Based on the similarities in surgical procedures, evaluation methods and the details in the reported outcomes (Tables 2 and 3), six studies out of all included studies were selected for further analyses since these studies had reported a mean change in nasal septum angle after LF-IO among all their participants [3, 18, 21, 24–26].

**Fig. 3:**
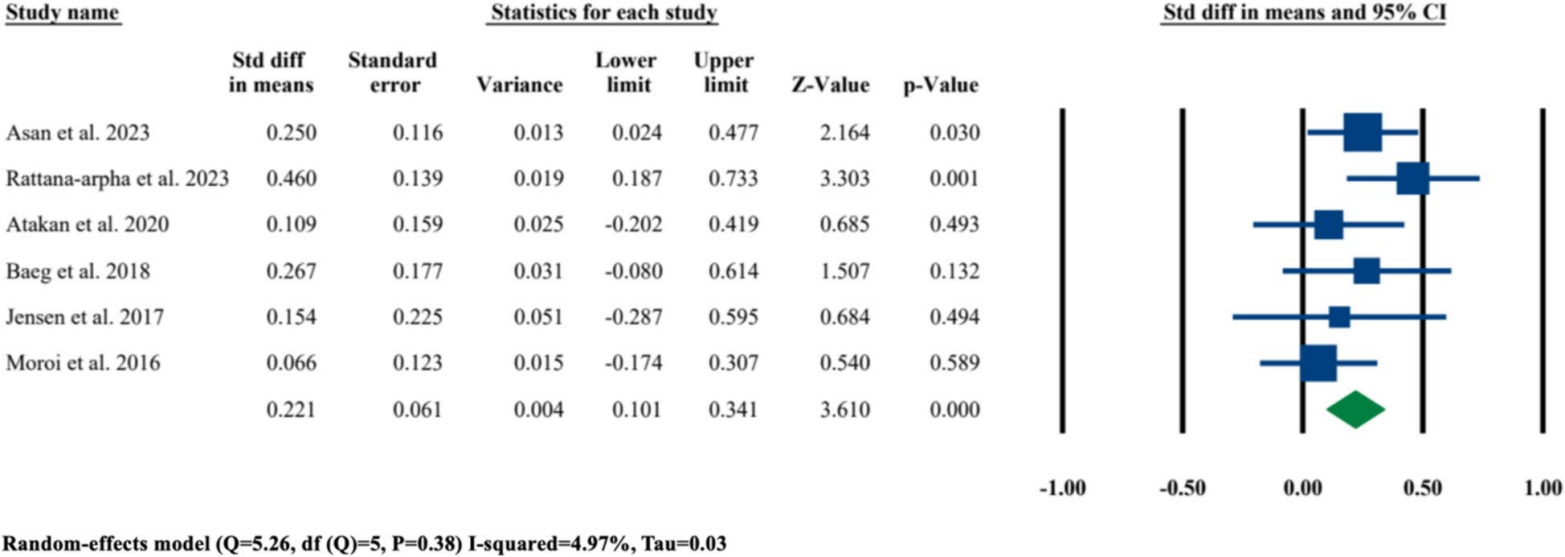
Standardized differences in means of nasal septum angle changes

Among these six selected studies, a meta-analysis was performed on the mean changes in nasal septum angle after LF-IO regardless of their surgical procedure (i.e., single-piece LF-IO, or segmental LF-IO), or evaluation method (i.e., CBCT, or CT, or cephalometry), or evaluation period. The results showed that the standardized difference in means of nasal septum angle changes among these six studies was statistically significant at 0.221° (95% CI 0.101–0.341, *P* = 0.000) (Fig. 3).

**Fig. 4:**
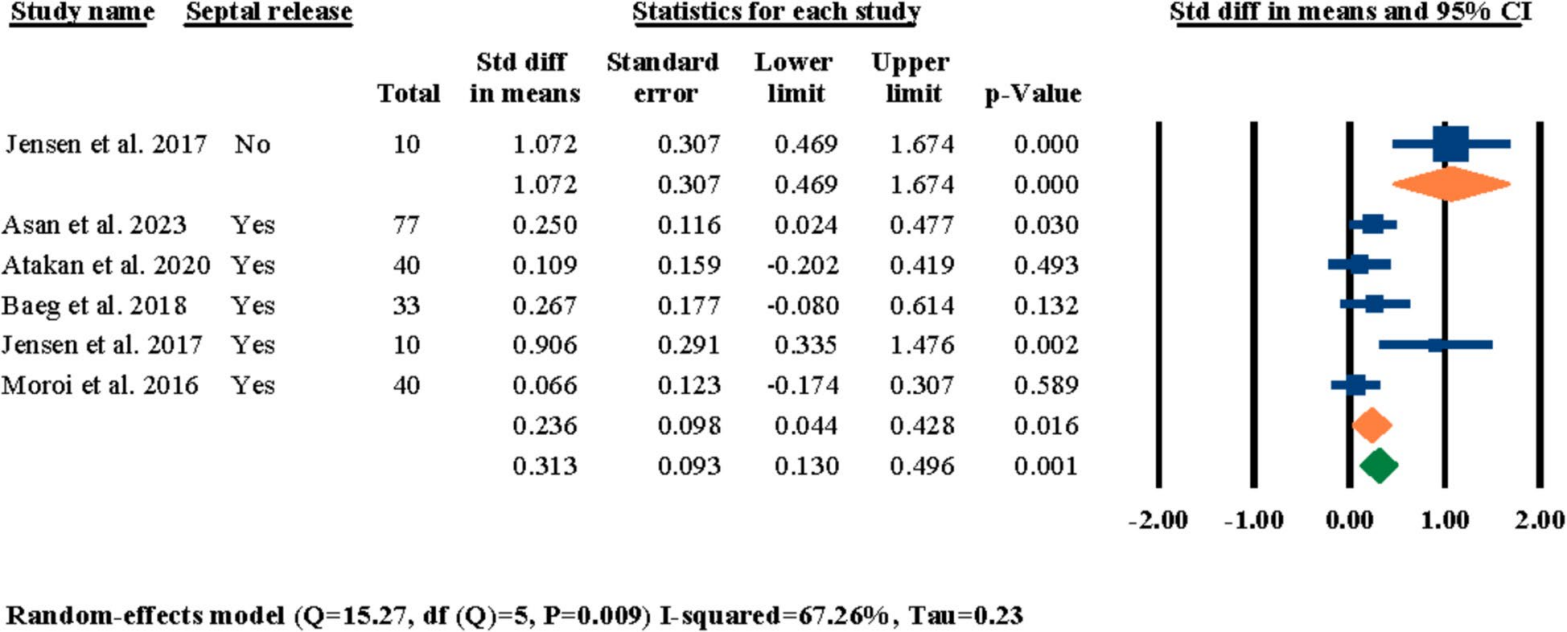
Standardized differences in means of nasal septum angle changes based on the release of the nasal septum

Out of these six selected studies, five of them had specified whether the nasal septum was released during the surgical procedure or not; the nasal septum was released in all patients in four studies [3, 21, 25, 26], while in Jensen et al.’s study, there were two groups of patients with ten members each and in one group nasal septum was released while in the other group it was not [24]. A meta-analysis was performed for these five studies and the results of standardized mean differences showcased a statistically significant standardized mean difference of 0.313° (95% CI 0.130–0.496, *P* = 0.001) in nasal septum angle changes after LF-IO (Fig. 4); the same analysis showed a statistically significant standardized mean difference of 0.236° (95% CI 0.044–0.428, *P* = 0.016) among studies that had released the nasal septum during LF-IO and a statistically significant standardized mean difference of 1.072° (95% CI 0.469–1.674, *P* = 0.000) in the study that did not release nasal septum during LF-IO.

**Fig. 5:**
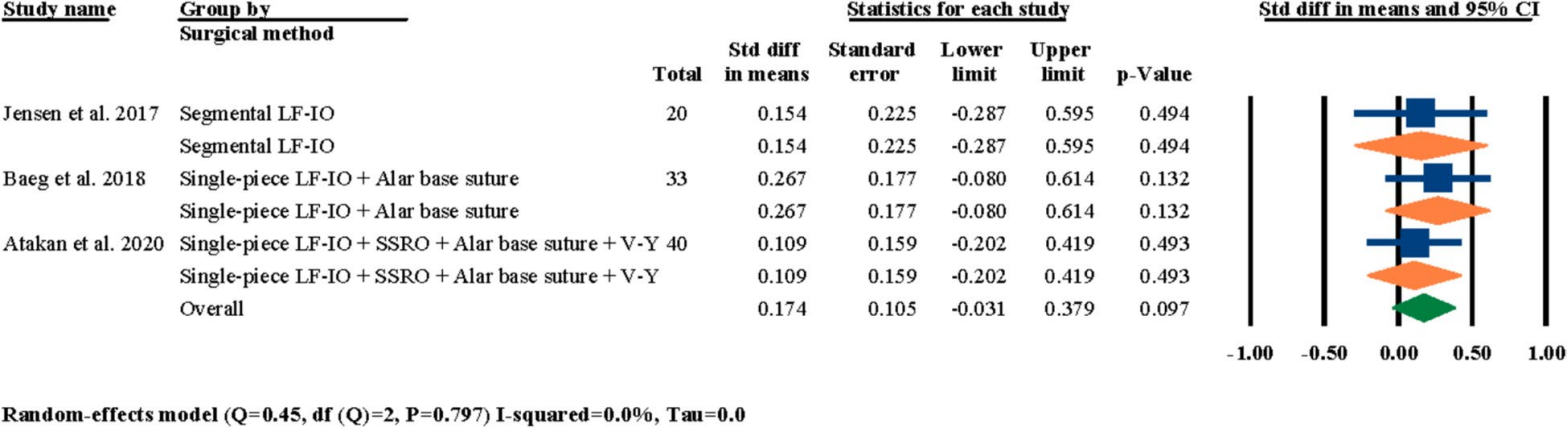
Standardized differences in means of nasal septum angle changes based on the type of surgical procedure

Regarding the influence of the type of surgical procedure on nasal septum angle changes after LF-IO, all of the six selected studies had reported their surgical procedure; however, in three of these studies [3, 18, 21] more than one surgical procedure was used and individual outcomes for different surgical procedures were not reported and an overall outcome of nasal septum angle changes for all patients had been reported; therefore, these three studies could not be included in this section of the meta-analysis that focuses on the influence of different surgical procedures. In the three remaining studies [24–26], a meta-analysis was performed to investigate the effect of the type of surgical procedure On nasal septum angle changes; the results showed statistically insignificant standardized mean differences at 0.174° (95% CI − 0.031–0.379, *P* = 0.097) (Fig. 5).

**Fig. 6:**
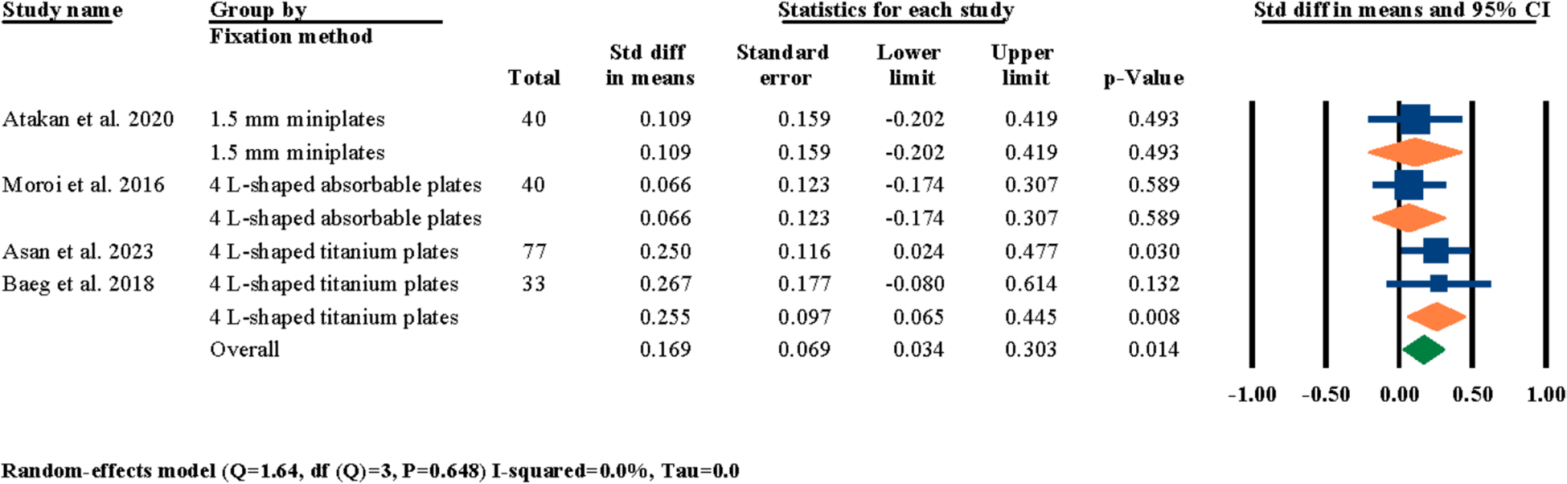
Standardized differences in means of nasal septum angle changes based on fixation method

Four of the selected studies had specified the skeletal fixation method of their LF-IO osteotomy procedure [3, 21, 25, 26]. Therefore, a meta-analysis was performed to investigate the effect of surgical fixation method On nasal septum angle changes after LF-IO; the standardized difference in means was statistically significant at 0.169° (95% CI 0.034–0.303, *P* = 0.014) (Fig. 6).

**Fig. 7:**
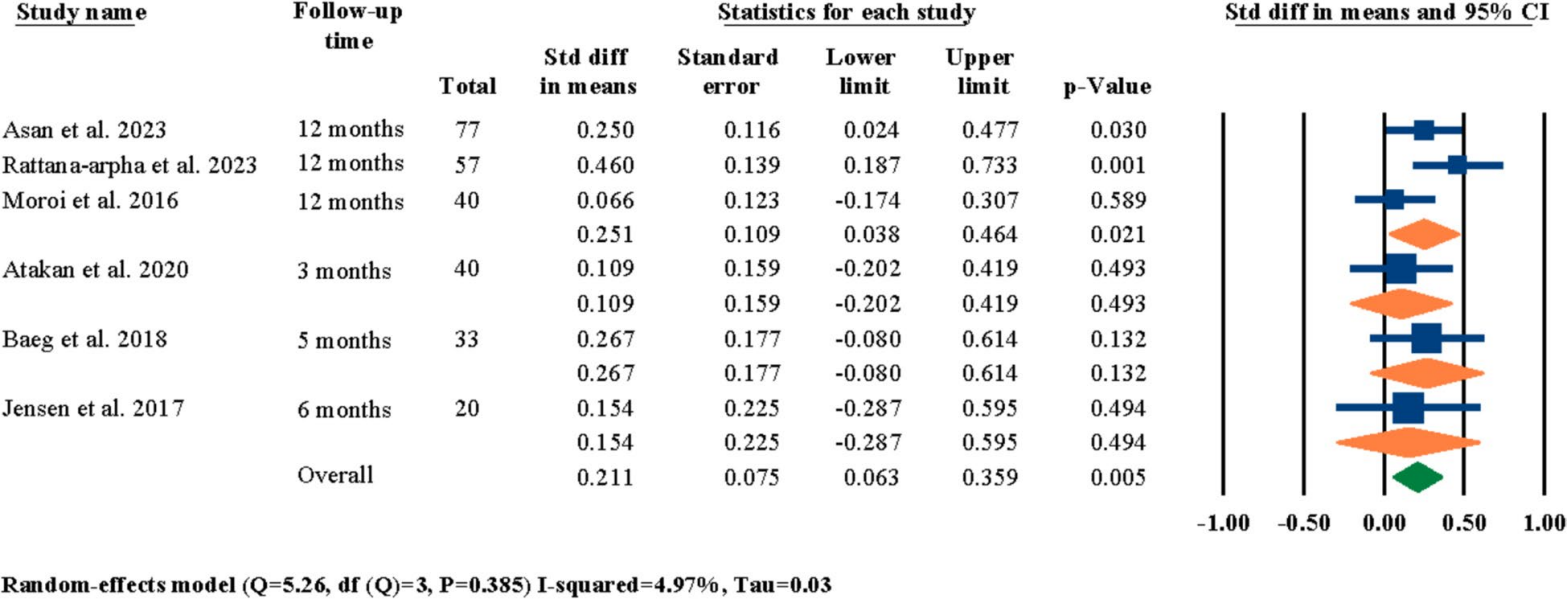
Standardized differences in means of nasal septum angle changes based on evaluation period

**Fig. 8:**
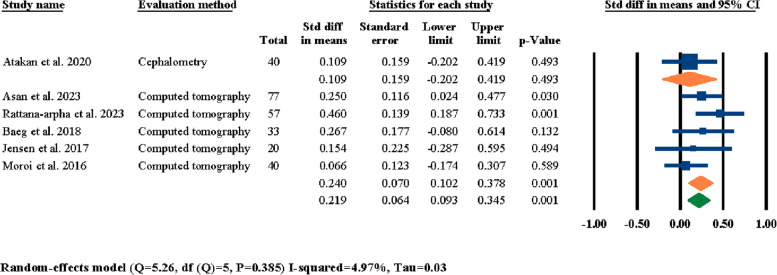
Standardized differences in means of nasal septum angle changes based on evaluation method

Another meta-analysis was conducted to investigate the influence of the time of evaluation period on reported outcomes of LF-IO on nasal septum changes based on the reported evaluation periods by each of the six selected studies (Table 3); the results showed statistically significant outcomes in terms of standardized differences in means of nasal septum angle changes at 0.211° (95% CI 0.063–0.359, *P* = 0.005) (Fig. 7). In addition, the effect of method of evaluation on reported outcomes of changes in nasal septum angle was also investigated. Four studies out of the six selected studies had reported their outcomes based on their CBCT findings [3, 18, 24, 25], one study used CT [21], and one study used cephalometry imaging [26] to evaluate nasal septum angle. Since both CBCT and CT radiographies use the same imaging methodologies and the frontal view of the head and face hard and soft tissue feature has the exact same detailing, these two radiographies were categorized under the same group as “computed tomography” and were treated equally for the meta-analyses. The reports showcased statistically significant standardized differences in means of nasal septum angle changes based On the type of radiographic imaging at 0.296° (95% CI 0.106–0.486, *P* = 0.002) (Fig. 8); the reports for the cephalometry subgroup were statistically insignificant at 0.109° (95% CI − 0.202–0.419, *P* = 0.493) while being statistically significant for the computed tomography subgroup at 0.408° (95% CI 0.167–0.648, *P* = 0.001) (Fig. 8).

#### Symmetry, direction and magnitude of maxillary movements

**Fig. 9:**
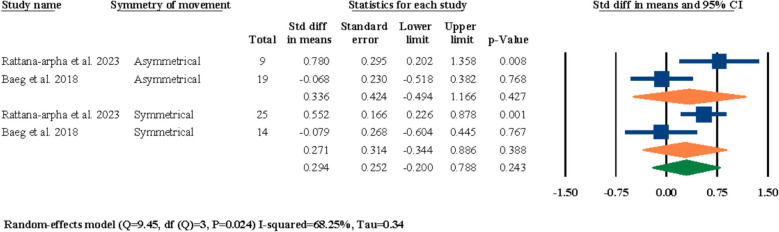
Standardized differences in means of nasal septum angle changes based on symmetry of movement

Out of the twelve included studies two of them had investigated the effects of symmetry/asymmetry of maxillary movements on post-surgical nasal septum angle changes [18, 25]. Both studies had two separate groups of patients who underwent symmetrical and asymmetrical LF-IO, regardless of the direction and/or magnitude of movement. The results of the meta-analysis were statistically insignificant for the overall evaluation at 0.294° (85% CI − 0.200–0.788, *P* = 0.243) and each of the asymmetrical and symmetrical subgroups at 0.336° (95% CI − 0.494–1.166, *P* = 0.427) and 0.271° (95% CI − 0.344–0.886, *P* = 0.388), respectively (Fig. 9).

**Fig. 10:**
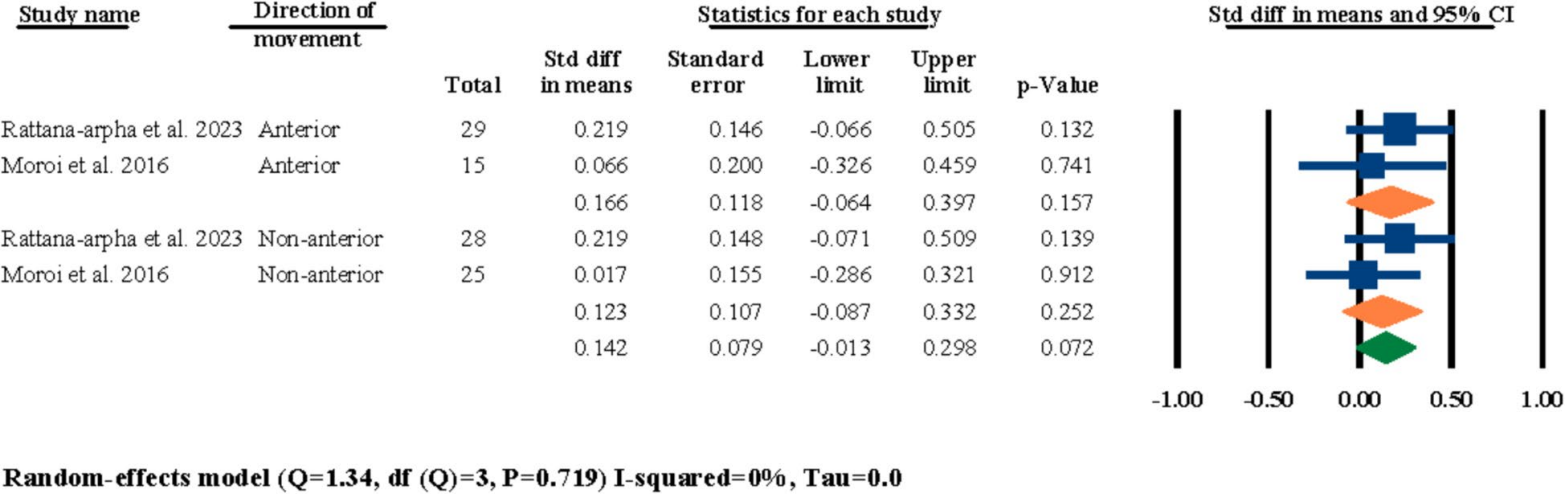
Standardized differences in means of nasal septum angle changes based on direction of movement

Two of the included studies had investigated the effects of the direction of the maxillary movements (i.e., anterior movement versus non-anterior movement) on post-surgical nasal septum angle changes [18, 21]. Both studies had two separate groups of patients based on the direction of the movement of their maxilla, regardless of the symmetry and/or magnitude of movement. The results of the meta-analysis were statistically insignificant for both of the anterior and non-anterior movement subgroups at 0.166° (95% CI − 0.064–0.397, *P* = 0.157) and 0.250° (95% CI − 0.275–0.776, *P* = 0.350) (Fig. 10). Furthermore, the overall evaluation for both groups was also statistically insignificant at 0.142° (95% CI − 0.013–0.298, *P* = 0.072) (Fig. 10).

**Fig. 11:**
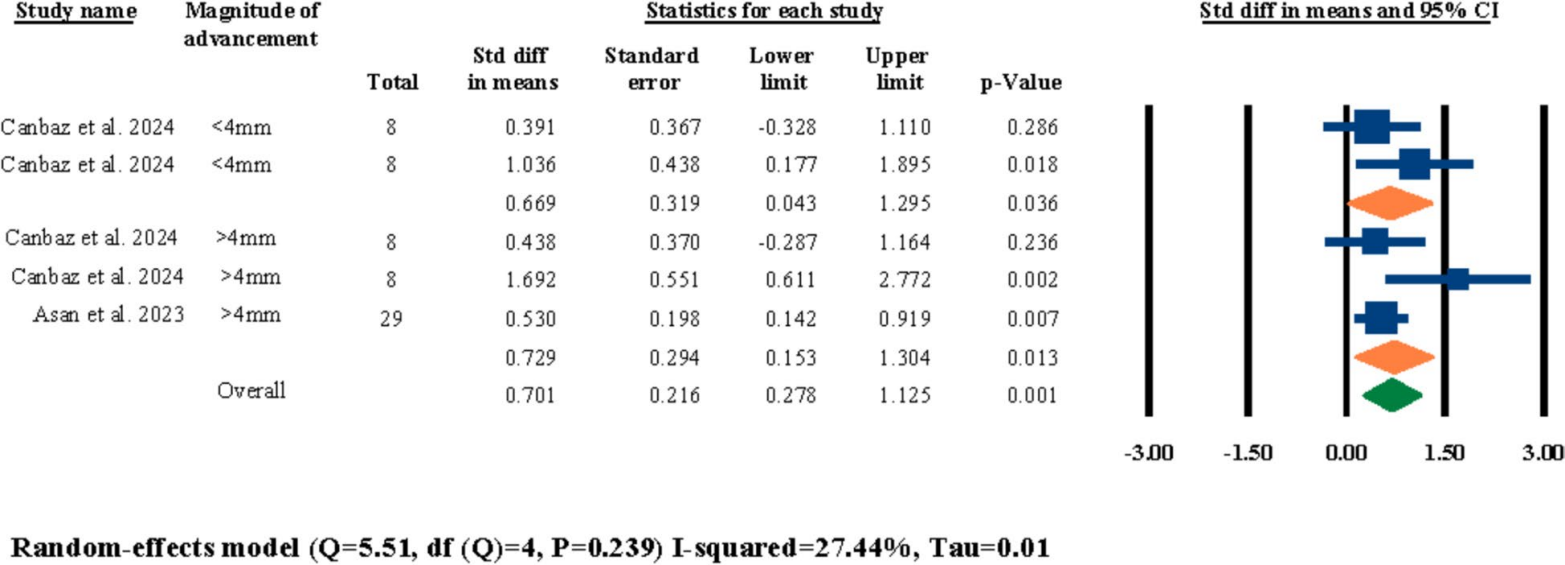
Standardized differences in means of nasal septum angle changes based on magnitude of advancement

**Fig. 12:**
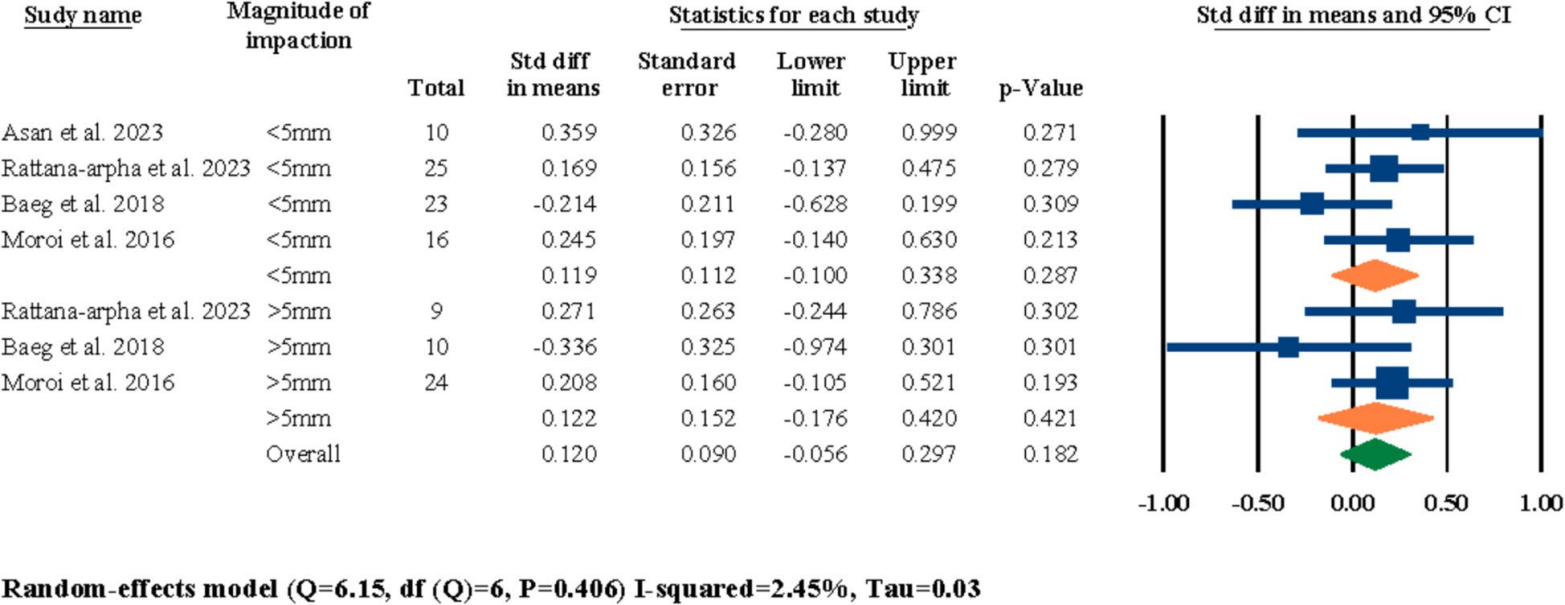
Standardized differences in means of nasal septum angle changes based on magnitude of impaction

Five out of the twelve included studies had investigated the effects of different amounts/magnitudes of maxillary impaction or advancement on post-surgical nasal septum angle changes [3, 18, 21, 25, 31]. Out of these five studies, only one of them had investigated and compared advancement movements against impaction movements [3], while the remaining four studies had only focused on one type of movement. Hence, two separate meta-analyses were executed; one for different magnitudes of advancement (Fig. 11) and one for different magnitudes of impaction (Fig. 12). The subgroups for each of the analyses were indicated based on the different categorizations of the selected studies, which fortunately did have similarities for both evaluations and proper analytical evaluations were feasible for both of the advancement and impaction meta-analyses. The results of the advancement movement meta-analysis were statistically significant for both of the “lower than 4 mm” and “more than 4 mm” advancement subgroups at 0.669° (95% CI 0.043–1.295, *P* = 0.036) and 0.729° (95% CI 0.153–1.304, *P* = 0.013), respectively (Fig. 11). Moreover, the overall evaluation for advancement movements were also statistically significant at 0.701 (95% CI 0.278–1.125, *P* = 0.001) (Fig. 11). In regard to the analytical evaluations for the impaction movements, the meta-analysis results for both of the “lower than 5 mm” and “more than 5 mm” subgroups were statistically insignificant at 0.249° (95% CI − 0.119–0.617, *P* = 0.185) and 0.005° (95% CI − 0.522–0.533, *P* = 0.985), respectively (Fig. 12). In addition, the overall meta-analysis evaluation for both subgroups were also statistically insignificant at 0.169° (95% CI − 0.133–0.471, *P* = 0.272) (Fig. 12).

#### Event rate of nasal septum deviation

**Fig. 13:**
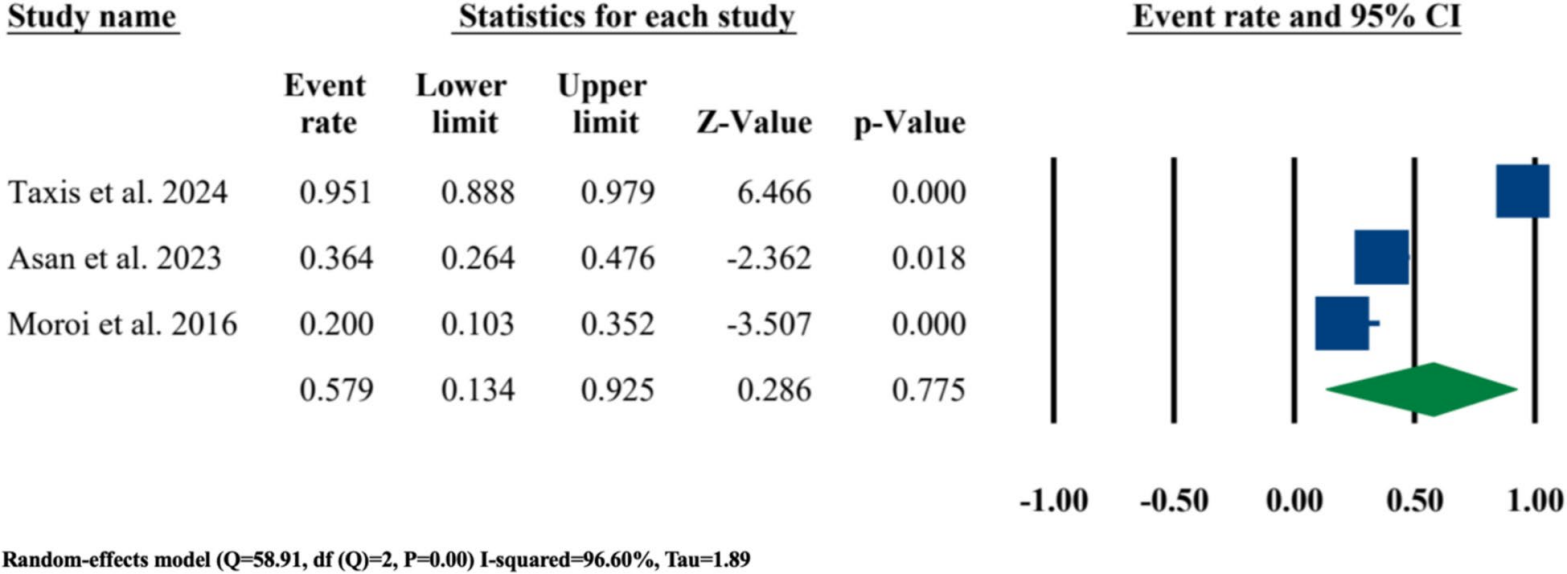
Event rate of nasal septum deviation

Three of the included studies had reported the actual number of patients that had a statistically significant change in their nasal septum angle after LF-IO and, subject to the opinion of the authors of each these three studies, they had also suffered from a significant clinical deviation in their nasal septum [3, 21, 32]. Hence, a meta-analysis was performed to investigate the event rate of nasal septum deviation in these three studies; the results showed an event rate of 0.579 (57.9%) (95% CI 0.134–0.925, *P* = 0.775) (Fig. 13).

#### Publication bias

**Fig. 14:**
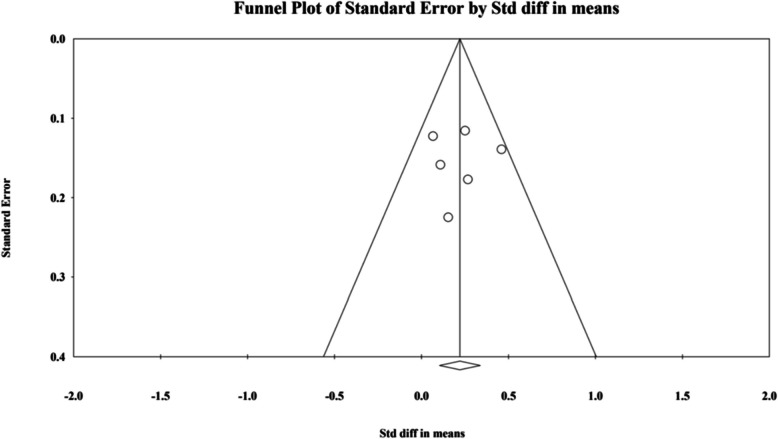
Funnel plot of standard error

The publication bias for the six studies [3, 18, 21, 24–26] that were selected for most of the analysis on septum angle changes after LF-IO was evaluated based on the Egger test, which showcased no evidence of bias (Fig. 14).

#### Alar base width

**Fig. 15:**
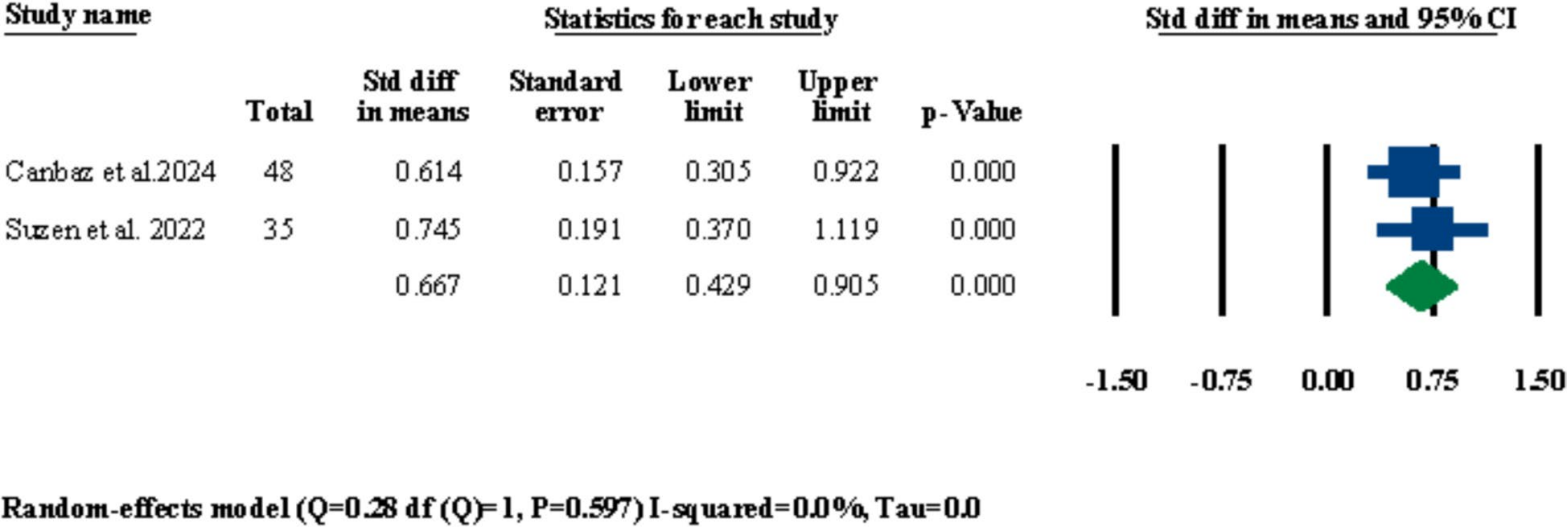
Standardized differences in means of alar base width changes

**Fig. 16:**
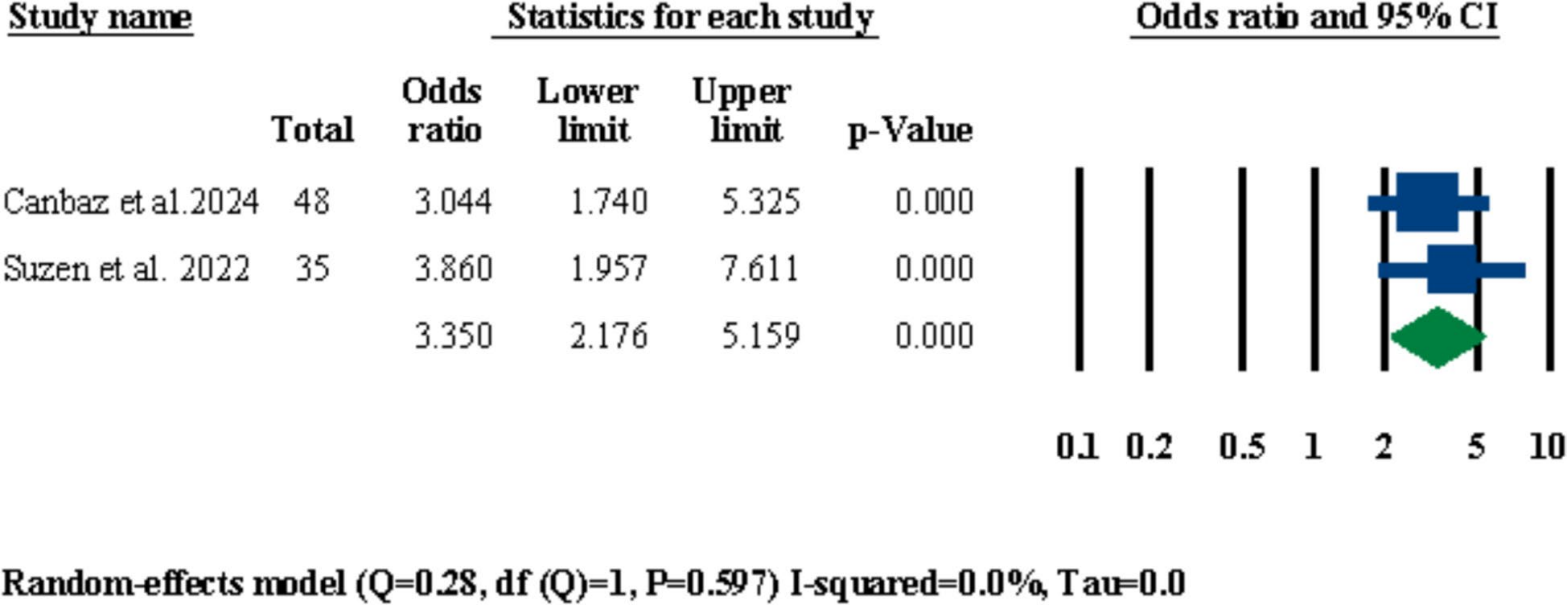
Odds ratio of alar base width changes

Out of the twelve included studies, there were two studies [29, 31] that had reported means in their patients’ alar base width before and after LF-IO. A meta-analysis was performed for these two studies to investigate significant changes in alar base width after LF-IO and the results were statistically significant both in terms of standardized differences in means and odds ratio at 0.667 mm (95% CI 0.429–0.905, *P* = 0.000) (Fig. 15) and 3.350 (95% CI 2.176–5.159, *P* = 0.000), respectively (Fig. 16).

#### Other outcomes

Along with nasal septum angle changes/deviation and alar base width that were reported in most studies, there were a variety of other factors regarding the health of the nose after LF-IO (Table 2). However, since these factors were mostly reported in one study each, they were not included in further analyses. One study reported the changes in their patients’ dimensions of the nasal airway area/space; Moroi et al. reported that right/left symmetry of the airway area in all of their cases were not significantly affected after LF-IO [21]. There were two studies that investigated the height and width of the nasal cavity; both studies reported significant decreases in the height of both right and left nasal cavities after LF-IO [26, 29]. Two studies reported the changes in nasolabial angle and nasal width after LF-IO [28, 31]; both studies reported that no significant changes occurred in nasolabial angle after LF-IO. However, regarding nasal width, Yamashsita et al. [28] reported no significant changes while Canbaz et al. [31] reported a significant increase in most of their cases. Two studies investigated the effects of LF-IO on nasal tip protrusion [28, 29]; Yamashsita et al. reported no significant changes [28], while Suzen et al. reported a significant decrease in nasal tip protrusion among their patients [29].

#### Quality assessment

**Fig. 17:**
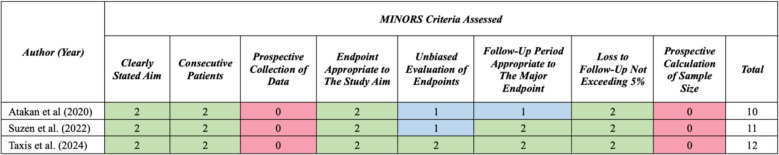
The MINORS assessment for non-comparative studies

**Fig. 18:**
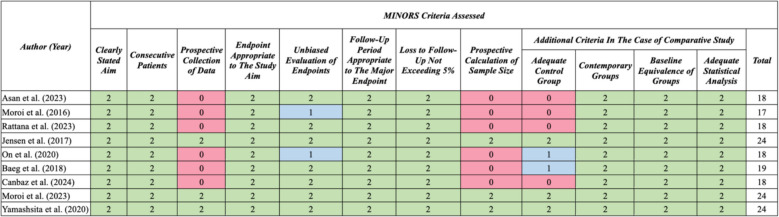
The MINORS assessment for comparative studies

The methodological index was used for both non-comparative [26, 29, 32] and comparative [3, 18, 21, 24, 25, 27, 28, 30, 31] studies. Figures 17 and 18 showcase the detailed ratings given to each study along with their total score. Among the three non-comparative studies, one of them had a high quality [32] based on its total score, while the other two showed a moderate quality in reporting [26, 29]. Out of the nine comparative studies, three of them had a high quality in reporting [24, 28, 30], while the remaining six all had a moderate quality in their reporting [3, 18, 21, 25, 27, 31].

## Discussion

Maxillary orthognathic surgeries utilizing LF-IO are so commonly and regularly performed since a lot of adult patients suffer from serious orthodontic issues that in most cases have a prominent and hard-to-ignore effect on their facial esthetics. These severe cases of orthodontic malocclusions combined with facial deformities cannot be treated with minimally invasive treatments alone and require major surgical interventions. When maxilla and its surrounding bony and cartilaginous structures, including the bony and cartilaginous septum, go through a major invasion and manipulation like LF-IO, it comes as no surprise that a variety of post-surgical soft tissue and hard tissue complications can be expected. As reported and discussed in various papers and books, the oral and maxillofacial surgeons that perform the orthognathic LF-IO release the nasal septum in most of their cases; even though there have been numerous benefits reported regarding the standard execution of LF-IO that was first introduced almost a century ago, it is worth noting that in the past couple of years a lot of surgeons have been investigating modified versions of LF-IO to minimize its post-surgical complications. One of the main complications that clinicians and researchers have been trying to combat is post-surgical nasal septum deviation. As mentioned before, since the nasal septum gets released from its inferior attachments to the maxilla bone during LF-IO, reattaching and fixing the nasal septum to its previous natural position is a surgical challenge that even if performed correctly could still cause post-surgical complications like septum deviation. The main reason that led to the design and execution of this systematic review was the fact that even though there are many reported cases of nasal septum deviations post LF-IO, to the best of the authors’ knowledge, there are no systematic reviews focused on investigating the rates and analytical reports of nasal septum deviations reported after orthognathic LF-IO. In order to cut back the bias of the post-surgical outcomes to the minimum, only studies were included that had performed the LF-IO on patients that did not have any congenital facial deformities or any major facial traumas, and had not previously received any kind of orthognathic and/or rhinoplasty surgeries. Moreover, in order to be able to report reliable reports, only studies that had reported their outcomes in rates, percentages, or further analytical assessments were included.

In the conventional approach of LF-IO, when maxilla is planned to be repositioned, both the bony and cartilaginous sections of the nasal septum will need to be dissected from the maxilla; vomer and the nasal crest of the maxilla will be removed from the maxilla equal to the total amount of the planned maxillary movement (i.e., both impaction and advancement). If surgeons miss these resections or fail to perform them correctly, buckling and distortion of the nasal septum, specifically the septal cartilage, will be highly expected [33]. However, the very point of this systematic review was to investigate the occurrence chances and incidence rates of nasal septum deviations after a conventional LF-IO has been performed correctly and according to the established guidelines and protocols. This decision was made based on the fact that a number of original studies reported shocking rates of nasal septum deviations after conventional LF-IO especially after maxillary impaction [3, 27], which contradicts the previous reports of a 1.6% incidence rate for nasal septum deviation after LF-IO by Kramer et al. in 2004 [34]. These deviations become serious burdens for patients when some of them will require secondary rhinoplasties to fix their nasal septum deviations. The exact incidence of nasal septum deviation has not been established by any researcher yet; even though Kramer et al. reported an incidence rate for nasal septum deviations, but they failed to provide a clear definition for nasal septum deviation. There are many challenges that contribute to the lack of these demographic data in the literature; there is an unsettling Heterogeneity among the opinions of different surgeons and researchers around the world regarding the definition of nasal septum deviation and its best diagnostic methodologies. In 2014, Aziz et al. conducted a systematic review to investigate the accuracy, sensitivity, and specificity of different measurement tools for the diagnosis of nasal septum deviation; it was concluded that even though different measurement methodologies come with their unique sets of benefits, the number of studies is still so limited to make any of these methods the gold standard of nasal septum deviation diagnosis [35]. Moreover, at best, these methods will be able to indicate abnormalities in the bony and cartilaginous nasal septum structures but they fail to indicate the severity of these abnormalities. Even if the nasal septum angle changes get properly diagnosed, there are contradicting reports in the literature in regard to which structural changes require to be surgically treated [36].

One of the key challenges of executing this systematic review and meta-analysis was the fact that not all of the included studies had utilized the same evaluation methodologies for measuring nasal septum angle and deviation after LF-IO; out of the twelve included studies, two of them had a rather unusual way of measuring nasal septum angle and deviation after LF-IO [28, 29], while the remaining ten included studies all had a clear and detailed description of their radiographic measurements and evaluations of nasal septum angle and deviation before and after LF-IO [3, 18, 21, 24–27, 30–32]. The radiographic measurement methodologies of these ten studies are all executed on the coronal section of the CT or CBCT scans and all can be categorized into two major groups: (1) In the first method, a line is drawn from the highest point of the Crista Galli to the lowermost point of the nasal septum at the transition to the maxilla (i.e., where vomer meets the nasal crest of Maxilla and the nasal crest of Palatine) which acts as the ideal midline that a perfectly straight nasal septum will fully align with this line, then a second line is drawn from the highest point of the Crista Galli to the most protruded/deviated point of the nasal septum (i.e., septal cartilage), and the angle made between these two lines at the reference point of Crista Galli indicates the nasal septum angle/deviation. It is worth noting that this angle will be at 0° in a perfectly straight and aligned nasal septum [18, 24, 26, 27, 31, 32]; (2) In the second method, initially a plane is drawn from connecting the left and right zygomatic-frontal sutures, then a second plane is drawn from the midsagittal suture of the hard palate, and the point that these two planes cross each other is marked. A line will be drawn from the marked point, which is located on the top of the bony nasal septum, to the most protruded/deviated point of the nasal septum (i.e., septal cartilage). A second line will be drawn from the lowermost point of the nasal septum to the most protruded/deviated point of the nasal septum (i.e., septal cartilage). The angle that is made between these two lines indicates the amount of nasal septum angle/deviation. In an ideal case, these two lines should perfectly align Onto each other and the angle between them would be at 180° [3, 21, 25, 30]. It is worth noting that out of the six studies that were selected for further meta-analysis on the changes in nasal septum angle after LF-IO (i.e., reported outcomes in mean average degrees), three of them followed the first mentioned method of measuring nasal septum angle/deviation [18, 24, 26], while the other three applied the second method [3, 21, 25]. Even though both nasal septum angle measurement methods have their own set of benefits and challenges, the literature suggests that the more standard way of measuring nasal septum angle/deviation, in terms of number of similar studies using this method, would be the first mentioned method; the angle that is made between the two lines of Crista Galli to the lowermost point of nasal septum and Crista Galli to the most protruded/deviated point of septal cartilage [37–39]. However, it goes without saying that the second method has not shown any real disadvantages compared to the first method and both methods can be easily relied on as long as they have been utilized and applied properly. As aforementioned, there were two different kinds of measuring methods for nasal septum angle/deviation among these 6 selected studies; however, they were still carried on for further analysis based on the mean difference in nasal septum angle reported in all of their patients; even though the two measurement methods have different standards for their ideal degrees (i.e., an ideal of 0° in the first method compared to an ideal of 180° in the second method), every single degree of change in the nasal septum angle/deviation has the same weight and value in both methods and they share that sentiment. Therefore, these six studies were selected for further analysis based on their reported mean changes in the nasal septum angle/deviation regardless of their nasal septum angle/deviation radiographic measurement methodologies [3, 18, 21, 24–26].

As showcased in the results section, a number of different meta-analyses were performed to indicate the significancy of standardized mean differences in post-surgical nasal septum angle changes based on different factors. First, the effects of LF-IO on nasal septum angle were analyzed and it was determined that the influence of LF-IO in causing post-surgical nasal septum angle changes was significant (Fig. 3). This finding indicates that regardless of the soft tissue measurements, the post-surgical changes of the nasal septum (i.e., the septal cartilage) angle measured through radiography was statistically significant. Next, the effects of releasing the septal cartilage from its surrounding bony compartments (i.e., ANS, and the nasal crest of the maxilla) during LF-IO were investigated and the results for both the overall evaluation and the two study groups were all statistically significant (Fig. 4). However, it is worth noting that the “no release” subgroup had only one study while the “release” subgroup had five studies. This finding is interesting since even the subgroup that did not surgically interact with the nasal septum (i.e., the septal cartilage) still reported significant mean changes in nasal septum angle of their patients. However, the authors of the study that had two separate study groups of “no release” and “release” have the subjective opinion that these angular changes were not significant in any of their study groups and that LF-IO, with and without nasal septum release, does not have a significant effect on nasal septum angle [24], while our analytical reports suggest otherwise. Next, the influence of different surgical procedures was analyzed on post-surgical nasal septum angle changes and the results were statistically insignificant for overall evaluations and each of the subgroups (Fig. 5); it is worth noting that all three subgroups had only one study in them and the fact that other studies could not be included in this analysis was due to the fact that even though they had different study groups based on their surgical methodologies, the results that they had reported was representative for all patients regardless of their surgical procedure; hence, they could not be selected for meta-analysis. The next meta-analysis was executed to investigate the effects of fixation methods on post-surgical nasal septum angle changes (Fig. 6); even though the overall results were statistically significant, two of the subgroups had only one study in them and both subgroups had statistically insignificant results, while only one subgroup had two studies and showed statistically significant results which was the “4 L-shaped titanium plates” (Fig. 6). In the next section of the meta-analyses, the radiographic evaluation methods and periods were investigated regarding post-surgical nasal septum angle changes (Figs. 7 and 8). For different evaluation periods, the overall evaluation was statistically significant; only the “12 months” subgroup had two studies and had statistically significant results while the “3 months,” “5 months,” and “6 months” subgroups each had one study and all three had statistically insignificant results. It is worth noting that even though some clinicians/researchers believe that evaluations conducted at 6 months post-surgery is considered long term, the authors of the current systematic review strongly believe that due to the complicated and invasive nature of LF-IO and its many influences On both the bony and cartilaginous sections of the nasal septum, evaluating the nasal septum angle through radiographic imaging at 12 months post-surgery must be a necessity. Regarding the different radiographic evaluation methods, the computed tomography subgroup (i.e., CT and CBCT) had six studies while the cephalometry group had only one study; the overall meta-analysis results for all selected studies were statistically significant; however, only the computed tomography subgroup showed statistically significant results (Fig. 8). The utilization of cephalometry imaging for the evaluation of nasal septum angle changes has been explored in relatively limited number of studies compared to computed tomography that seem to be the gold standard.

In the next section of the meta-analyses executed in this systematic review, the effects of symmetry of movement, direction of movement, and different magnitudes of impaction and advancement on post-surgical nasal septum angle changes were investigated. For the symmetry/asymmetry evaluations, two studies were selected [18, 25]; both studies had two study groups of symmetrical and asymmetrical maxillary movements during LF-IO and both studies reported statistically insignificant differences between their two study groups in regard to the post-surgical nasal septum angle changes. In the meta-analysis executed for these two studies, the overall results and the results of both of the subgroups (i.e., symmetry, and asymmetry) were all statistically insignificant (Fig. 9). This finding showcases a clear void in the literature in regard to the lack of adequate studies investigating the effects of symmetry/asymmetry in maxillary impaction/advancement on nasal septum angle changes. Both of these selected studies had surgically interrupted/released the septal cartilage in all of their patients, and given the statistically insignificant results reported by both the original studies and the meta-analysis of this systematic review, it can be interpreted that the surgical manipulation of nasal septum seems to have a more prominent effect than the symmetry of the maxillary movement. Next, for the evaluations on the direction of the movement, two studies were selected for meta-analysis [18, 21]; both studies had compared the outcomes of anterior versus non-anterior movements of the maxilla during LF-IO and had reported statistically insignificant results between their two study groups. The overall meta-analysis results and the results for both subgroups were all statistically insignificant (Fig. 10). Both of these studies had surgically manipulated/released the nasal septum in all of their patients, and while these analytical outcomes are valuable, it is clear that these two studies alone are not adequate for reaching a firm conclusion on the effects of maxillary direction of movement on post-surgical nasal septum angle changes and more studies are required in the literature. Regarding the magnitude of maxillary advancement and its effects on nasal septum angle changes, two studies were selected [3, 31]; Asan et al. reported that the magnitude of advancement has no significant effect on nasal septum angle changes [3], while Canbaz et al. reported that advancements lower than 4 mm has a worst impact On nasal septum angle than advancements bigger than 4 mm. Moreover, Canbaz et al. reported that in general, chances of post-surgical nasal septum angle changes are higher in cases of total impaction than advancement [31]. The results of the meta-analysis performed on these two studies were statistically significant for overall evaluation and both of the subgroups individually (Fig. 11). However, the current available data in the literature suggests that the effects of different magnitudes of maxillary movement on nasal septum angle cannot be concluded due to the inadequate published studies. In regard to the evaluations on the magnitude of impaction and its effects on nasal septum angle, four studies were selected for meta-analysis [3, 18, 21, 25]; all four studies had two separate study groups of “less than 5 mm” and “more than 5 mm” of maxillary impaction and all four of them reported insignificant differences between the outcomes of their two study groups and concluded that the magnitude of maxillary impaction cannot predict post-surgical nasal septum angle changes. The results of the meta-analysis for the overall evaluation and each of the two subgroups were all statistically insignificant, which aligns with the findings of all of the four selected studies that there are no adequate data to solidify a correlation between the magnitude of maxillary impaction and nasal septum angle changes (Fig. 12).

One of the meta-analyses performed in this systematic review was regarding the event/incidence rate of nasal septum deviation; as mentioned before, there are still no objectively reliable incidence rates reported in the literature. Out of the twelve included studies in this systematic review, only three of them had reported actual numbers of their patients that, in their opinion, suffered from post-surgical nasal septum deviation [3, 21, 32]. With an event rate of 0.579 (57.9%) (95% CI 0.134–0.925, *P* = 0.775), it could be concluded that even though only three studies could have been included in this analysis, the incidence rate of nasal septum deviation after conventional LF-IO is way higher than what previous studies have reported in the literature (i.e., the 1.6% incidence rate reported by Kramer et al.) (Fig. 13).

### Less invasive surgical procedures

Out of the twelve included studies in this systematic review, there is a study that has tried to focus on a relatively new way of operating LF-IO without any interactions with the nasal septum (i.e., the septal cartilage) [28]. Yamashsita et al. had two study groups; one group underwent conventional single-piece LF-IO while in the second group, the single-piece LF-IO was performed in a sub-spinal manner. This modified surgical procedure has one major difference with conventional LF-IO which is cutting the maxilla beneath the ANS and the septal cartilage in contrast with the conventional method which cuts the maxilla above the ANS and resects the septal cartilage from its surrounding bony compartments (i.e., ANS, the nasal crest of the maxilla, and vomer), by doing this Yamashsita et al. believe that the risks of nasal septum angle changes will be significantly reduced; however, the authors have failed to properly investigate the nasal septum (i.e., the septal cartilage) angle through radiographic evaluations and have focused on extraoral soft tissue landmarks; therefore, their reports could not have been included in the meta-analyses of this systematic review. However, in a study by Mommaerts et al., they concluded that sub-spinal LF-IO has no superiority to conventional LF-IO in terms of minimizing nasal tip changes and/or prevailing control over the columello-labial angle [40]. It is also worth noting that Yamashsita et al. reported that based on the questionnaires that all of their patients filled, the number of patients that were satisfied with their nasal and facial esthetics after LF-IO were bigger in the sub-spinal group [28].

### Post-surgical precautions

In a study by Moroi et al., the effects of using nostril retainers after the completion of conventional single-piece LF-IO was investigated; they had two study groups each with thirty patients, both groups underwent conventional single-piece LF-IO but Only One group used a fixed appropriately sized nostril retainer for 7 days post-surgery [30]. All of their nostril retainers were obtained from KOKEN Co. (Tokyo, Japan) and each patient was given a proper size of the retainer based on the Mold Guide of KOKEN Co. (Tokyo, Japan); all retainers were fixed in patients’ noses with the help of tapes. The main focus of this study was to gain better symmetry for the left and right nostrils after LF-IO. Moroi et al. reported that the nasal cavity ratios (i.e., the ideal ratio in a perfectly symmetrical nose between the two nostrils would be at 1) at 1 year post-surgery was significantly higher in the retainer group, indicating that the two nostrils had higher rates of symmetry in the retainer group [30]. Moreover, it was reported that there was a significant difference in anterior nasal septum between the two groups; the authors reported that the no-retainer group suffered more from post-surgical nasal septum deviation. It is no mystery that in conventional orthognathic LF-IO, not only the nasal septum is at a high risk of being affected by deformities due to the stress generated during the separation of the septal cartilage and bone fragment movements, the nasotracheal intubation used for almost all cases of general anesthesia in LF-IO can also cause significant deformities during both intubation and extubation [41–43]. These nostril retainers have also been previously used to improve the morphology of nasal alar and/or nasal cavity after the surgical treatment of nasal bone fractures [44, 45]. Moroi et al. concluded that even though these supportive nostril retainers can prevent significant deviation and deformation of the nasal septum in the short term, the shape maintenance effect of these retainers in the long term is not as significant and relapses of minor septum deformities might be expected [30]. Hence, Moroi et al. have suggested that maybe long-term relapse of minor nasal septum deformities could be combatted with longer utilization of these retainers (i.e., longer than 7 days), and future studies must focus on the appropriate period of using nostril retainers.

### Alar base cinch suture

In orthognathic surgeries, mainly LF-IO-based orthognathic surgeries, the predictability of bony movements/changes and fixation process post-surgery has been investigated and reported in a number of human studies with positive insights [46, 47]. Despite the favorable and predictable outcomes of skeletal changes post LF-IO, the effects that soft tissue structures might suffer from LF-IO osteotomies have been less investigated and hence cannot be properly predicted [48]. Regardless of how successful and predictable the bony changes and movements occur after LF-IO, as long as there is no clear predictability established for the changes that happen to the soft tissue structures of the nose (i.e., the septal cartilage, the nasal tip, the alar base width, the alar base symmetry) after LF-IO, the satisfaction of both the surgeons and patients cannot be properly predicted. Unfortunately, to the best of the authors’ knowledge, the influence of different directions and amounts of orthognathic maxillary movements on alar base width has not been properly investigated in the literature [49]. It has been reported that the transoral vestibular approach of conventional LF-IO leads to the widening of alar base width because of the detachment of the muscles that hold the nasal structure to their correct position in midface [50]. Releasing facial muscles from their nasolabial roots and anterior nasal spine gives them the freedom to retract laterally, which leads to the flaring, rising, and widening of the nasal base, which in most cases happens asymmetrically [51, 52]. To combat the high possibility of significant changes to the alar base width, two suture techniques have been widely used [53, 54]: (1) The classic technique, also referred to as the internal technique, was first introduced in 1980 by Millard et al. which is widely applied in patients with cleft lip to correct nasal defects; however, it was not until 1982 that Collins and Epker used this technique in patients undergoing orthognathic LF-IO [55, 56]; (2) In 2002, Shams and Motamedi reported a new external technique to prohibit the undesirable changes to the width of the alar base after LF-IO [57]. There have been many modifications and alterations made to the conventional alar base cinch suture methods; in a scoping review executed by Rauso et al. published in 2022, all of the different methods of alar base cinch sutures were categorized into four types and it was concluded that further human clinical studies are required to compare these methods against each other in regard to long-term stability of alar base width and nasal tip positioning[58].

Out of the twelve included studies in the current systematic review, ten of them had performed alar base cinch sutures for all of their patients (Table 3) which only three of them had reported the changes in alar base width in their patients after LF-IO [28, 29, 31]. Two out of these three studies had reported the mean changes in the width of the alar base among all of their patients measured in millimeters [29, 31]; both of these studies had used the conventional alar base cinch suture technique established by Shams and Motamedi [57] which is the Type I method based on the Rauso et al. classification [58]. The results of the meta-analyses performed for these two studies showcased a significant increase in the width of the alar base with a standardized mean difference of 0.667 (95% CI 0.429–0.905, *P* = 0.000) (Fig. 15). Yamashsita et al. had compared the changes in alar base width after LF-IO between their two study groups (i.e., conventional single-piece LF-IO, and sub-spinal single-piece LF-IO) and reported that the sub-spinal group had a significantly lower increase in their alar base width (mean: 0.54 ± 0.67 mm) compared to the conventional group (mean: 1.99 ± 1.40 mm) [28]. Various reports in the literature overwhelmingly agree that performing alar base cinch suture is necessary after LF-IO; however, given the different types of alar base cinch sutures and the lack of conclusive data on the long-term differences between these methods, further human clinical studies are required to reach a definitive protocol for choosing the right type of alar base cinch suture for each type of LF-IO based on the numerous factors that can lead to significant differences in the outcomes of the procedure (e.g., in regard to the magnitude of impaction or advancement, single-piece or segmental LF-IO, anterior movement or non-anterior movement of the maxilla). Moreover, Yamashsita et al.’s study showed that sub-spinal LF-IO leads in significantly lower increases in the width of the alar base compared to conventional LF-IO [28]; this shows that maybe not interacting with ANS and the nasal septum (i.e., the septal cartilage) in the first place might be more helpful than finding the right treatment after conventional LF-IO to prohibit significant increases in alar base width.

### Limitations and suggestions

1. The six studies selected for meta-analysis in this systematic review were categorized into two separate methods that both use frontal section views in computed tomography. Even though they were all used for analysis and their methodologies were discussed in details, it would be ideal to have an established radiographic methodology so that all researchers will be able to easily conduct similar studies that will be compatible for meta-analyses. Experts in the field of oral and maxillofacial radiology can report comparative analyses of these two measurement methodologies and report which method has the highest sensitivities and lowest chances of operator-based errors. Or, the least that could happen is for researchers to clearly define and even name these two major radiographic evaluation methods to make more analyses and comparisons feasible in the future. Moreover, the evaluation periods reported in the studies varied from 3 to 12 months post-surgery; even though some clinicians and researchers think that six months post-surgery is considered long-term, the literature suggests that it takes at least 12 months for the hard and soft tissue compartments of both the maxilla and nasal septum to fully Heal and showcases all of their potential major and minor deformities and relapses. Therefore, it is highly suggested that future studies try to follow the safest and most reliable protocols in terms of evaluation methods and periods; computed tomography evaluations before and 12 months after surgery with previously established nasal septum angle measurement methodologies. Given the fact that some reports in the literature have shown minor and/or major relapses in LF-IO procedures beyond the first 12 months post-surgery [59], surgeons and researchers are highly encouraged to have follow-up sessions with their patients for years after LF-IO.
2. A considerable number of included studies had failed to properly report adequate data regarding the rationale behind their surgeries (e.g., maxillary forward/downward/upward movements, different skeletal malocclusions, maxillary expansions) and their surgical procedures. It is highly suggested that authors report all of the following data in their clinical studies since each factor can have meaningful impact on the outcomes of the surgery: (A) single-piece or segmental LF-IO; (B) conventional LF-IO or sub-spinal LF-IO or sub-nasal LF-IO; (C) with or without the surgical resection of the septal cartilage from its surrounding bony compartments; (D) with or without alar base cinch suture (and if alar base cinch suture was performed, the exact type of the suture must also be specified); (E) the symmetry/asymmetry, direction of the maxillary movement, the exact type of maxillary manipulation (i.e., impaction or advancement), and the magnitude of maxillary movements in all directions.
3. Out of the twelve included studies, except for one clinical trial, all of them had a cohort study design. Even though the limitations for performing a proper randomized clinical trial is understandable, it is highly suggested that future studies try to execute randomized clinical trials; it goes without saying that in order to reach firm conclusions on the long-term comparative outcomes of different types of LF-IO combined with different kinds of alar base cinch sutures, a number of randomized clinical trials must be executed. Moreover, newly discussed post-surgical precautions like nostril retainers must also be further-explored and investigated in randomized study designs in order to get one step closer to minimizing post-surgical nasal septum deformities in the clinic.
4. The most crucial limiting factor for this systematic review, was the fact that to the best of the authors’ knowledge, there are still no established guidelines or protocols for the diagnosis of nasal septum deviation. Moreover, the very definition of “deviation” has not been properly established either; even studies that had reported detailed mean and standard deviation numbers for their nasal septum angle before and after surgery, have different standards sometimes biases in considering certain degrees of changes in the nasal septum angle as nasal septum “deviation.” Furthermore, some researchers solely focus on the extraoral landmarks of patients’ nose and midface to rule out post-surgical nasal deformities. On top of all of that, patients’ satisfaction might not be aligned with the analytical outcomes of their radiographic evaluations, and one of the challenging aspects of orthognathic LF-IO is the fact that since it heavily manipulates the hard and soft tissue compartments of the nose and midface with unpredictable degrees of extraoral manifestations, it is crucial to help patients have reasonable expectations from their surgeries. The authors of this systematic review strongly believe that experts in oral and maxillofacial surgery and head and face esthetics must definitely try to come up with clear definitions of nasal septum deviation; more importantly a distinct threshold must be introduced in order to indicate what amounts of nasal septum degree changes must be considered as “deviation.” Also, as mentioned in the first limitation section, even a gold standard for radiographic evaluation methodology has not been established yet; the ideal will be if researchers establish their indicating thresholds in a specific radiographic evaluation methodology so that researchers/clinicians from all around the world will have a standard manner in their evaluation methodologies.

## Conclusions

LF-IO significantly increases the nasal septum angle and in most cases leads to deviation. Maxillary advancement movements during LF-IO cause significant increases in nasal septum angle. LF-IO causes significant widening in the alar base of the nose. There are still no established guidelines on the definition and diagnosis of nasal septum deviation other than each surgeon’s subjective opinion. There are still no established guidelines for the correct radiographic evaluation methodology of nasal septum angle after LF-IO. Future studies must focus on defining nasal septum deviation, finding a radiographic gold standard for its diagnosis, and instituting a threshold for the exact amount of degrees changed in nasal septum angle that can be considered deviation. Moreover, less-invasive procedures such as sub-spinal LF-IO must be further investigated. Patients undergoing orthognathic LF-IO must be informed regarding the possible adverse effects of LF-IO on nasal structures.

## Supplementary Information

Supplementary Material 1. Supplementary Table 1. Extracted data

## Data Availability

All of the extracted data and reported analyses have been reported and showcased in details in the main text of this systematic review and meta-analysis.

## Abbreviations

LF-IO: Le Fort I osteotomy
SSRO: Sagittal split ramus osteotomy
BSSRO: Bilateral sagittal split ramus osteotomy
CT: Computed tomography
CBCT: Cone beam computed tomography
ANS: Anterior nasal spine
