# Supplementary material for: Nasal septum deviation after orthognathic Le Fort I osteotomy: a systematic review and meta-analysis
Source: Maxillofac Plast Reconstr Surg. 2025 Oct 9;47(1):29. doi: 10.1186/s40902-025-00483-8 (PMC12508338; doi:10.1186/s40902-025-00483-8)
Supplement: Supplementary file 1 — Supplementary Material 1. Supplementary Table 1. Extracted data [file 40902_2025_483_MOESM1_ESM.docx]

**Supplementary Table 1.** Extracted data.

| ***A) Author (Year)***  ***B) Study Design*** | ***A) Number of Patients***  ***B) Patients’ Gender (M:F)***  ***C) Patients’ Age*** | ***Study Groups*** | ***A) Surgery Rationale***  ***B) Exclusion Criteria*** | ***A) Surgical Intervention***  ***B) Cartilage Manipulation***  ***C) Anesthesia***  ***D) Fixation Method*** | ***A) Study Variables***  ***B) Method(s) of Assessment (Evaluation Periods)*** | ***Results and Conclusions*** |
| --- | --- | --- | --- | --- | --- | --- |
| A) Asan et al. (2023) (3)  B) Retrospective cohort | A) 77  B) 33:44  C) Mean: 22.3 ± 6.1 / Range: 17-46 | A) Maxillary advancement (4.90 ± 1.59 mm, median: 5 (2.0-9.0 mm)) (n = 29) (Group 1)  B) Maxillary impaction (2.84 ± 1.39 mm, median: 2.50 (1.0-8.0)) (n = 10) (Group 2)  C) Simultaneous maxillary impaction and advancement (n = 38) (Group 3)  **** Note:***  Nasal septum was released in all cases | A) Maxillary vertical and sagittal excess or deficiency (Class II and III malocclusions)  B) History of septoplasty or rhinoplasty  before orthognathic surgery; asymmetrical impactions or  maxillary elongation; congenital deformities  such as cleft lip and palate; posttraumatic deformities | A.1) Single-piece LF-IO + alar base cinch suture (n = 9)  A.2) Single-piece LF-IO + BSSRO + alar cinch suture (n = 68)  B.1) Cartilage reduction (n = 27)  B.1.1) Group 1 (n = 1)  B.1.2) Group 2 (n = 7)  B.1.3) Group 3 (n = 19)  C) Nasotracheal intubation  D) 4 L-shaped titanium plates | A) Nasal septum angle/deviation  B) CBCT scan (T0 and T12) | 1. Nasal septum angle changes at T12:  Decreased angle observed in 28 cases (12 males and 16 females) (36.4%): 2/28 only Le Fort I and 26/28 Le Fort I + BSSRO  2. The average of nasal septum angle:  2.1. Significant increase in all patients: T12 (168.7°) >> T0 (166.2°) (P = 0.031)  2.2. Significant increase in Group 1: T12 (169.80°) >> T0 (167.30°) (absolute differences: 2.5°, inter quartile range: 3.95°) (P = 0.008)  2.3. Group 2: T12 (170.25°) ≈ T0 (166.70°) (absolute differences: 1.2°, inter quartile range: 5.20°) (P = 0.285)  2.4. Group 3: T12 (165.15°) ≈ T0 (164.70°) (absolute differences: 0.8°, inter quartile range: 6.98°) (P = 0.717)  3. In total, 28 patients had septum deviation at T12  3. Nasal septal deviation:  8/27 (29.62%) of cases with and 20/50 (40%) of cases without cartilage reduction had nasal septum deviation at T12; however, none of them were clinically visible  4. Nasal septum deviation:  Group 3 (n = 20) (52.6%) >> Group 1 (n = 2) (20.0%) ≈ Group 2 (n = 6) (20.7%) (P = 0.014)  5. The average of nasal septum angle at T12 had no correlation with patients’ age, gender and surgery type (P > 0.05)  6. There were no correlations between nasal septum angle and the magnitude of maxillary advancement/impaction  7. Both maxillary advancement (P = 0.049) and impaction magnitudes (P = 0.001) were significantly higher in cases with cartilage reduction  8. Mean magnitude of maxillary advancement and impaction were at 4.90 ± 1.59 mm (2.0-9.0 mm) and 2.84 ± 1.39 mm (1.0-8.0 mm), respectively  **** Conclusions:***  Forward and upward movement of maxilla can worsen nasal septum deviations. |
| A) Moroi et al. (2016) (21)  B) Retrospective cohort | A) 40  B) 14:26  C) Mean: 24.6 / Range: 16-49 | A) Based on the amount of maxillary impaction:  - A.1. Less than 4 mm (n = 16) (Group A.1.)  - A.2. More than 5 mm (n = 24) (Group A.2.)  B) Based on the direction of movement:  - B.1. Anterior movement (n = 15) (Group B.1.)  - B.2. Non-anterior movement (n = 25) (Group B.2.)  C) Based on impaction symmetry:  - C.1. Symmetry group (less than 3 mm difference between the impaction of the right and left sides) (n = 20) (Group C.1.)  - C.2. Asymmetry group (more than 4 mm difference between the impaction of the right and left sides) (n = 20) (Group C.2.)  **** Note:***  Nasal septum was released in all cases | A.1) Bi-maxillary asymmetry (n = 5)  A.2) Bi-maxillary asymmetry + mandibular prognathia (n = 18)  A.3) Mandibular prognathia (n = 11)  A.4) Open bite (n = 5)  A.5) Mandibular prognathia + open bite (n = 1)  B) Not specified | A.1) Single-piece LF-IO + alar base cinch suture (n = 1)  A.2) Single-piece LF-IO + BSSRO + alar base cinch suture (n = 39)  B) Not specified  C) Not specified  D) 4 L-shaped absorbable plates | A) Nasal septum angle (anterior, middle, and posterior angles), nasal septum deviation, and the dimension and health of the airway area  B) CT scan (T0, T1/4, T12) | 1. Insignificant nasal septum deviation was observed in 8 (20%) cases  2. The occurrence rate of airway area deformities and nasal septum deviation at T0, T1/4 and T12:  2.1. Group A.1. ≈ Group A.2. (P = 0.2290)  2.2. Group B.1. ≈ Group B.2. (P = 0.6857)  2.3. Group C.1. ≈ Group C.2. (P = 0.7025)  3. The difference in nasal septum angle overtime in all groups:  3.1. Anterior: T0 (89.3° ± 3.1°) ≈ T1/4 (89.0° ± 7.9°) ≈ T12 (87.8° ± 8.8°)  3.2. Middle: T0 (87.9° ± 5.7°) ≈ T1/4 (89.0° ± 7.9°) ≈ T12 (88.3° ± 6.3°)  3.3. Posterior: T0 (89.6° ± 2.7°) ≈ T1/4 (89.4° ± 2.9°) ≈ T12 (89.6° ± 2.8°)  4. Nasal septum angle changes amongst all cases was insignificant over between T0, T1/4, and T12:  between subjects (P = 0.5052) and within subjects (P = 0.6464)  5. Nasal septum angle changes in different groups:  5.1. Group A.1.: T0 (86.7° ± 6.0°) ≈ T12 (88.3° ± 6.9°)  5.2. Group A.2.: T0 (89.6° ± 5.0°) ≈ T12 (88.5° ± 5.5°)  5.3. Group B.1.: T0 (86.1° ± 6.9°) ≈ T12 (87.5° ± 6.4°)  5.4. Group B.2.: T0 (89.0° ± 4.6°) ≈ T12 (88.9° ± 6.3°)  **** Conclusions:***  Conventional LF-IO does not lead to significant changes in nasal septum angle and/or the right/left symmetry of the airway area in all 40 cases. |
| A) Atakan et al. (2020) (26)  B) Retrospective cohort | A) 40  B) 17:23  C) Mean: 20.52 ± 4.4 / Range: not specified | Not specified  **** Note:***  Nasal septum was released in all cases | A) Skeletal class III malocclusion  B) Congenital craniofacial deformity, previous facial trauma,  craniofacial syndromes,  naso‑maxillary surgery and orthognathic surgery with  inferior positioning of maxilla | A) Single-piece LF-IO + BSSRO + alar base cinch suture + V-Y closure (n = 40)  B) Not specified  C) Nasotracheal intubation  D) A 1.5 mm mini plates system | A) Nasal cavity height and width, nasal base angle, and nasal septum deviation  B) Posterior-anterior cephalometry and lateral cephalometry (T0 and T3) | 1. Changes of nasal septum deviation between T0 and T3:  0.26° ± 2.39° (P = 0.496, 95% CI)  2. Changes of nasal base angle between T0 and T3:  0.2° ± 2.77° (P = 0.964, 95% CI)  3. Nasal cavity height changes at T3:  3.1. Right side (–2.04 ± 3.13 mm) (P = 0.000, 95% CI)  3.2. Left side (–1.99 ± 3.21 mm) (P = 0.000, 95% CI)  **** Conclusions:***  The effect of LF-IO + BSSO on nasal septum deviation was insignificant, however, the nasal cavity height significantly decreased for both right and left sides. |
| A) Rattana‑arpha et al. (2023) (18)  B) Retrospective cohort | A) 57  B) 17:40  C) Mean: 30.32 / Range: 24-42 | A) Based on the amount of maxillary impaction:  - A.1. More than 5 mm (n = 9)  - A.2. Less than 5 mm (n = 25)  - A.3. Not specified (n = 23)  B) Based on the direction of movement:  - B.1. Anterior movement (n = 29)  - B.2. Non-anterior movement (n = 28)  C) Based on impaction symmetry:  - C.1. Symmetry impaction (n = 25)  - C.2. Asymmetry impaction (n = 9)  - C.3. Not specified (n = 23)  D) Maxillary impaction vs. inferior repositioning:  - D.1. Impaction (n = 34)  - D.2. Inferior repositioning (n = 8)  - D.3. Not specified (n = 15)  E) Type of LF-IO:  - E.1. Single-piece osteotome (n = 50)  - E.2. Segmental osteotomy (n = 7)  **** Note:***  The releasing of nasal septum was not specified | A) Not specified  B) Craniofacial anomaly, post-traumatic facial deformity, and history of septorhinoplasty or turbinoplasty | A.1) Single-piece LF-IO + alar base cinch suture (n = 50)  A.2) Segmental LF-IO + alar base cinch suture (n = 7)  B) Not specified  C) Not specified  D) Not specified | A) Nasal septum angle (both at the Nasion and Ostium levels)  B) CBCT scan (T0 and T12) | 1. Nasal septum angle changes:  1.1. At Nasion level:  A Significant increase was observed at T12 amongst all cases; T0 (1.72° ± 2.73°) << T12 (2.41° ± 3.31°) (mean difference: 0.69° ± 1.47°) (P = 0.001)  1.1.1. Increased angle: 29.83% (n = 17); from 3.65° ± 2.89° at T0 to 6.12° ± 2.75° at T12 (mean difference: 2.47° ± 1.61°)  1.1.2. Decreased angle: 5.26% (n = 3); from 7.17° ± 3.01° at T0 to 6.33° ± 2.57° at T12 (mean difference: −0.83° ± 0.58°)  1.1.3. No change: 64.91% (n = 37)  1.2. At Ostium level:  A significant increase was observed at T12 amongst all cases; from 3.26° ± 2.96° at T0 to 4.04° ± 2.96° at T12 (mean difference: 0.77° ± 2.14°) (P = 0.006)  1.2.1. Increased angle: 49.12% (n = 28); from 3.46° ± 2.40° at T0 to 5.59° ± 1.89° at T12 (mean difference: 2.13° ± 2.08°)  1.2.2. Decreased angle: 17.55% (n = 10); from 6.50° ± 2.01° at T0 to 4.95° ± 2.52° at T12 (mean difference: −1.55° ± 1.61°)  1.2.3. No change: 33.33% (n = 19)  1.3. There were no significant differences between each of the subgroups regarding their changes in nasal septal angle both at the Nasion and Ostium levels at T12.  2. Nasal septum angle changes in different groups:  2.1. A.1.: T0 (1.67° ± 2.05°) ≈ T12 (2.50° ± 3.30°)  2.2. A.2.: T0 (2.10° ± 3.10°) ≈ T12 (2.66° ± 3.47°)  2.3. B.1.: T0 (1.90° ± 2.92°) ≈ T12 (2.60° ± 3.37°)  2.4. B.2.: T0 (1.54° ± 2.57°) ≈ T12 (2.21° ± 3.30°)  2.5. C.1.: T0 (1.96° ± 3.11°) ≈ T12 (2.60° ± 3.65°)  2.6. C.2.: T0 (2.06° ± 2.04°) ≈ T12 (2.67° ± 2.65°)  2.7. D.1.: T0 (1.99° ± 2.84°) ≈ T12 (2.62° ± 3.37°)  2.8. D.2.: T0 (1.25° ± 1.75°) ≈ T12 (2.00° ± 2.22°)  3. Nasal septum angle changes in different surgical methods:  3.1. Single-piece LF-IO: T0 (1.90° ± 2.85°) ≈ T12 (2.56° ± 3.42°)  3.2. Segmental LF-IO: T0 (0.43° ± 1.13°) ≈ T12 (1.36° ± 2.32°)  **** Conclusions:***  Nasal septal angle was significantly increased after LF-IO, however, differences in maxillary movement directions and segmentations had no significant effect on nasal septal angle. |
| A) Jensen et al. (2017) (24)  B) Prospective cohort | A) 20  B) 9:11  C) Mean: 20.5 / Range: 14-51 | 1. With intraoperative releasing of the nasal septum (n = 10)  2. Without intraoperative releasing of the nasal septum (n = 10) | A) Transverse maxillary deficiency of more than 5 mm hypoplasia  B) Previous orthodontic maxillary expansion; syndromal  patients (including clefts), history of maxillary or nasal  septum surgery; or mental retardation | A) Segmental LF-IO (n = 20)  B) Not specified  C) Nasotracheal intubation  D) Not specified | A) Nasal septum deviation (evaluated by measuring the angle obtained between the traced median reference line and the intersection line between the  superior orbital rim)  B) CBCT scan (TS, and T6) | 1. Nasal septum angle:  From TS to T6:  Mean: 0° / range: –0.62° to 0.62° (P = 0.5, 95% CI)  2. Nasal septum deviation:  No significant changes at T6 in all cases; the mean differences in nasal septum deviation from T0 to T6 was at 0.83° ± 0.71° and 0.83° ± 0.60° for the patients with intraoperative nasal septum releasing and without intraoperative nasal septum releasing, respectively.  **** Conclusions:***  LF-IO does not have any significant effect on nasal septum angle/deviation. There is no need for intraoperative nasal septum releasing. |
| A) On et al. (2020) (27)  B) Retrospective cohort | A) 28  B) 16:12  C) Mean: 21.81 / Range: > 16 | 1. Total impaction group (n = 13); Mean: 1.5 mm, range: 0.5-2 mm  2. Anterior elongation (n = 15);  Mean: 1.6 mm  range: 1.0-3.0 mm  **** Note:***  Nasal septum was released in all cases | A) Maxillary vertical excess or deficiency  B) craniofacial anomaly; post-traumatic facial deformity; history of  septorhinoplasty or turbinoplasty. | A) Single-piece LF-IO + BSSRO + alar base cinch suture (n = 28)  B) The cartilaginous part of nasal septum was resected to avoid deviation  C) Not specified  D) 4 L-shaped titanium plates | A) Nasal septum deviation (a total deviation index was calculated by summing the deviation indices of Nasion, crista galli, and the most  anterior point of the sphenoid sinus)  B) CBCT scan (T0 and T12) | 1. Nasal septum deviation from T0 to T12:  1.1. Total impaction group:  Significant increase in the total nasal septum deviation index: from 5.45 mm to 6.43 mm (P < 0.01)  1.2. Anterior elongation group:  No significant changes in any of the deviation indices (P > 0.05)  2. The amount of maxillary surgical forward movement was positively correlated with both the changes in the Nasion plane (r = 0.422, P < 0.05) and the total deviation index (r = 0.398, P < 0.05)  **** Conclusion:***  LF-IO + BSSRO can worsen nasal septum deviation especially in the posterior side of nasal septum |
| A) Baeg et al. (2018) (25)  B) Retrospective cohort | A) 33  B) 17:16  C) Mean: 24.1 / Range: not specified | A) Based on maxillary impaction:  A.1. Less than 4 mm (n = 23)  A.2. More than 5 mm (n = 10)  B) Based on horizontal movement:  B.1. Horizontal movement (n = 13)  B.2. No horizontal movement (n = 20)  C) Based on the symmetry of the movement on the left and right sides of the maxilla:  C.1. Symmetrical movement (n = 14)  C.2. Asymmetrical movement (n = 19)  D) Based on total impaction:  D.1. Total impaction (n = 4)  D.2. Non total impaction (n = 29)  **** Note:***  Nasal septum was released in all cases | A) Maxillary vertical excess or deficiency  B) Not specified | A) Single-piece LF-IO + alar base cinch suture (n = 33)  B) Nasal septum cartilage was separated and resected  C) Not specified  D) L-shaped titanium plates | A) Nasal septum angle  B) CBCT scan (T0 and T5) | 1. Nasal septum angle:  1.1. There were no significant changes in any of the groups and in all patients from T0 to T5 (P = 0.135)  1.2. Nasal septum angle increased in 10 cases; mean: 1.42° (0.12° - 4.19°)  1.3. Nasal septum angle decreased in 23 cases; mean: 1.47° (0.16° - 4.38°)  2. Nasal septum angle changes in each subgroup:  2.1. Group A.1.: from 10.7° at T0 to 10.4° at T5 (main decrease: 0.3°) (P = 0.315)  2.2. Group A.2.: from 10.7° at T0 to 10.0° at T5 (main decrease: 0.7°) (P = 0.315)  2.3. Group B.1.: from 10. 2° at T0 to 9.9° at T5 (main decrease: 0.3°) (P = 0.397)  2.4. Group B.2.: from 10.9° at T0 to 10.3° at T5 (main decrease: 0.6°) (P = 0.397)  2.5. Group C.1.: from 10.4° at T0 to 9.8° at T5 (main decrease: 0.6°) (P = 0.771)  2.6. Group C.2.: from 11.0° at T0 to 10.6° at T5 (main decrease: 0.4°) (P = 0.771)  2.7. Group D.1.: from 7.9° at T0 to 7.7° at T5 (main decrease: 0.2°) (P = 0.424)  2.8. Group D.2.: from 11.0° at T0 to 10.5° at T5 (main decrease: 0.5°) (P = 0.424)  **** Conclusions:***  LF-IO does not cause any significant changes in nasal septum angle. |
| A) Canbaz et al. (2024) (31)  B) Retrospective cohort | A) 48  B) 19:29  C) Mean: not specified / Range: > 18 | Based on the amount of maxillary advancement, impaction, and downward movement:  1. More than 4 mm advancement and less than 1.5 mm of impaction (n = 8)  2. Less than 4 mm advancement and less than 1.5 mm of impaction (n = 8)  3. More than 4 mm advancement and more than 1.5 of impaction (n = 8)  4. More than 4 mm advancement and more than 1.5 of downward movement (n = 8)  5. Less than 4 mm advancement and more than 1.5 of impaction (n = 8)  6. Less than 4 mm advancement and more than 1.5 of downward movement (n = 8)  **** Note:***  Nasal septum was released in all cases | A) Maxillary deficiency  B) age under 18 years, history of maxillofacial trauma, previous orthognathic surgery, previous rhinoplasty/septoplasty, type 4 (S-shaped) septum deviation, craniofacial deformities such as cleft lip/palate | A) Single-piece LF-IO + alar base cinch suture (n = 48)  B) Not specified  C) Nasotracheal intubation  D) Titanium plates | A) Nasal septum angle (the angle between the line from the anterior point of the nasal  spine (ANS) to the crista galli and the line from the crista galli to the tip  of the deviated nasal septum), nasal septum deviation volume, nasolabial angle, nasal width, and alar base width  B) CBCT scan (T0 and T6) | 1. Nasal septum angle:  1.1. Significantly increased in Group 3:  From 10.84° ± 5.75° at T0 to 12.75° ± 5.29° at T6 (P = 0.002)  1.2. Significantly increased in Group 5:  From 11.43° ± 2.34° at T0 to 12.56° ± 2.37° at T6 (P = 0.022)  1.3. Insignificant differences from T0 to T6 in Groups 1, 2, 4 and 6:  1.3.1. Group 1: from 9.18° ± 5.38° at T0 to 9.41° ± 5.24° at T6 (P = 0.255)  1.3.2. Group 2: from 11.08° ± 4.94° at T0 to 11.38° ± 4.91° at T6 (P = 0.305)  1.3.3. Group 4: from 11.94° ± 4.97° at T0 to 12.53° ± 4.43° at T6 (P = 0.098)  1.3.4. Group 6: from 9.73° ± 5.58° at T0 to 9.95° ± 5.88° at T6 (P = 0.247)  1.4. There were no significant differences between the 6 groups at T0 and T6 (P = 0.875 and P = 0.610, respectively)  2. Nasal septum deviation volume:  Significantly increased in Groups 2, 3 and 5 (P = 0.049, P = 0.007, and P = 0.026, respectively)  3. Differences in nasal septum angle (from T0 to T6) (ΔSDA) and differences in nasal septum deviation volume (from T0 to T6) (ΔSDV) compared within different groups:  3.1. ΔSDA and ΔSDV significantly increased in Group 3 (P = 0.001 and P = 0.011, respectively)  3.2. Group 6 showed significantly lowest ΔSDV values (P = 0.011)  3.3. Significant positive correlation between ΔSDA and ΔSDV values in Groups 2, 3, 4 and 5.  3.4. Significant positive correlation between ΔSDA and ΔSDV values in all cases ( r = 0.710, P < 0.001)  4. Soft tissue evaluations of alar base and nasal width (CBCT):  4.1. The differences of alar base and nasal width at T6 between different cases were significant (P < 0.001 and P = 0.017, respectively)  4.2. The differences of alar base and nasal width at T6 were significantly different between Group 1 and Group 5  **** Conclusions:***  The amount of maxillary surgical movement did not make a significant difference in nasal septum deviation. However, maxillary impaction can significantly increase septum deviation angle and volume. Both alar base width and nasal width increased after LF-IO. |
| A) Suzen et al. (2022) (29)  B) Retrospective cohort | A) 35  B) 12:23  C) Mean: 24.45 ± 6.52 / Range: not specified | Not specified  **** Note:***  Nasal septum was released in all cases | A) Not specified  B) Multi-piece Le Fort I osteotomy, inferior repositioning  of the maxilla, congenital deformities such  as cleft lip and palate, and previous history of trauma  and rhinoplasty | A) Single-piece LF-IO + alar base cinch suture (n = 35)  B) Not specified  C) Nasotracheal intubation  D) Four 2.0 mm miniplates | A) The angle between the longitudinal axis of the right and left  nostrils (aRLN), the angle of columella with the long axis of  right and left nostrils (aRCN (right) and aLCN (left)), alar base width, alar width, nasal tip protrusion, right nostril height and width, and left nostril height and width  B) CT scans (T0 and T6) | 1. aRLN:  1.1. A significant increase in the aRLN angle was observed from T0 (56.17° ± 18.2°) to T6 (69.51° ± 20.34°) (P = 0.000)  1.2. Significant correlation between maxillary impaction and aRLN (P = 0.032); an increase of 3.34° in aRLN for 1 mm maxillary impaction was  observed (P = 0.003, adjusted R^2^ = 0.21)  2. aRCN:  A significant increase in the aRCN angle was observed from T0 (30.26° ± 7.83°) to T6 (34.94° ± 7.94°) (P = 0.000)  3. aLCN:  A significant increase in the aLCN angle was observed from T0 (31.49° ± 7.39°) to T6 (36.82° ± 7.54°) (P = 0.000)  4. Alar base width:  A significant increase in alar base width was observed from T0 (29.47 ± 4.32 mm) to T6 (32.38 ± 3.21 mm) (P = 0.000)  5. Alar width:  5.1. A significant increase in alar width was observed from T0 (35.46 ± 3.88 mm) to T6 (38.43 ± 3.55 mm) (P = 0.000);  5.2. Every 1 mm of maxillary impaction increased alar width by about 0.37 mm (P = 0.023, adjusted R^2^ = 0.12)  6. Nasal tip protrusion:  A significant decrease in nasal tip protrusion was observed from T0 (20.98 ± 1.92 mm) to T6 (20.39 ± 1.77 mm) (P = 0.022)  7. Right nostril height:  A significant decrease in right nostril height was observed from T0 (13.59 ± 1.93 mm) to T6 (12.98 ± 2.04 mm) (P = 0.009)  8. Right nostril width:  A significant increase in right nostril width was observed from T0 (6.42 ± 1.50 mm) to T6 (7.14 ± 1.88 mm) (P = 0.001)  9. Left nostril height:  A significant decrease in left nostril height was observed from T0 (13.75 ± 2.21 mm) to T6 (12.92 ± 2.14 mm) (P = 0.002)  **** Conclusions:***  Following LF-IO, there was a significant  increase in alar base width, alar width, and angle between the longitudinal axis of the left and right nostrils, while nasal tip protrusion was significantly decreased. |
| A) Moroi et al. (2023) (30)  B) Retrospective cohort | A) 60  B) 15:45  C) Mean: 23.9 ± 6.3 / Range: 17-31 | A) Patients who used a nostril retainer (attached and fixed to the nasal alar base) for 7 days post-surgery (Group retainer) (n = 30);  - Mean maxillary advancement: 1.36 ± 1.33 mm  - Mean of elevation at anterior right was 2.43 ± 2.45 mm, at anterior left was 2.30 ± 2.09 mm, at posterior right was 3.17 ± 1.99 mm, and at posterior left was 2.77 ± 1.79 mm  B) Patients who did not use a nostril retainer for 7 days post-surgery (Group no-retainer) (n = 30);  - Mean of maxillary  Advancement: 0.73 ± 1.05 mm  - Mean of elevation  at anterior right was 2.27 ± 1.71 mm, at anterior left was  2.37 ± 2.07 mm, at posterior right was 2.43 ± 1.72 mm, and at posterior left was 2.53 ± 2.08 mm  **** Note:***  Nasal septum was released in all cases | A) Not specified  B) History of trauma in the oral and maxillofacial region; history of orthognathic  surgery; history of cleft lip and palate; history of maxillofacial syndrome; history of rhinoplasty or nasal septoplasty; posterior and inferior maxillary movement of LF-IO  fragments; and maxillary elevation of 6 mm or more | A) Single-piece LF-IO + alar base cinch suture (n = 60)  B) Nasal septum cartilage was separated and resected  C) Nasotracheal intubation  D) Not specified | A) Nasal septum angle (anterior, middle and posterior)  B) CT (T0 and T12) | 1. There was a significant difference in the amount of advancement between the retainer and no retainer groups (P = 0.035)  2. Nasal septum angle:  2.1. Significant difference in the posterior nasal septum angle between the two groups at T0 (P = 0.029, 95% CI); 175.8° ± 3.1° in Group no-retainer and 173.6° ± 4.0° in Group retainer  2.2. No significant differences in the anterior and middle nasal septum angles between the two groups at T0:  - Anterior nasal septum angle: 173.4° ± 4.6° in Group no-retainer and 175.0° ± 3.9° in Group retainer (P = 0.135, 95% CI)  - Middle nasal septum angle: 170.8° ± 6.3° in Group no-retainer and 169.4° ± 5.7° in Group retainer (P = 0.364, 95% CI)  2.3. Significant difference in the anterior nasal septum angles between the two groups at T12 (P = 0.019, 95% CI); 164.8° ± 11.7° (Group retainer) >> 156.9° ± 13.5° (Group no-retainer)  2.4. No significant differences in the middle and posterior nasal septum angles between the two groups at T12:  - Middle nasal septum angle: 166.2 ± 8.8° in Group no-retainer and 166.1° ± 11.6° in Group retainer (P = 0.988, 95% CI)  - Posterior nasal septum angle: 173.7° ± 12.4° in Group no-retainer and 174.0° ± 4.4° in Group retainer (P = 0.89, 95% CI)  2.5. Significant difference in anterior nasal septum angle between T0 and T12 in Group retainer (P < 0.001, 95% CI); 175° ± 3.9° (T0) >> 164.8° ± 11.7° (T12)  2.6. No significant differences in the middle and posterior nasal septum angles from T0 to T12 in Group retainer:  - Middle nasal septum angle: 169.4° ± 5.7° at T0 and 166.1° ± 11.6° at T12 (P = 0.106, 95% CI)  - Posterior nasal septum angle: 173.6° ± 4.2° at T0 and 174° ± 4.4° at T12 (P = 0.504, 95% CI)  2.7. Significant difference in anterior nasal septum angle between T0 and T12 in Group no-retainer (P < 0.001, 95% CI); 173.3° ± 4.6° (T0) >> 156.9° ± 13.5° (T12)  2.8. Significant difference in middle nasal septum angle between T0 and T12 in Group no-retainer (P = 0.002, 95% CI); 170.8° ± 6.3° (T0) >> 166.2° ± 8.8° (T12)  2.9. No significant difference in posterior nasal septum angle between T0 (175.8° ± 3.1°) and T12 (173.7° ± 12.4°) in Group no-retainer (P = 0.346, 95% CI)  **** Conclusions:***  Deformation of the nasal septum can be prevented by retainer treatment following LF-IO. |
| A) Yamashsita et al. (2020) (28)  B) Retrospective cohort | A) 39  B) 6:33  C) Mean: 23.8 / Range: 16-49 | 1. Single-piece LF-IO + BSSO + alar base cinch suture + V-Y closure (Group 1) (n = 19)  2. Sub-spinal single-piece LF-IO + BSSO + alar base cinch suture + V-Y closure (Group 2) (n = 20)  **** Note:***  The nasal septum was only released in Group 1; nasal septum and ANS were not manipulated in Group 2 | A) Anterior repositioning of the maxilla  B) History of congenital facial deformity or midfacial surgery | A) Single-piece LF-IO + BSSRO + modified alar base cinch suture + V-Y closure (n = 39)  B) Not specified  C) Not specified  D) Not specified | A) Nasolabial angle (columella-soft tissue nasion-labrale superior), nasal tip angle (soft tissue nasion-pronasale-subnasale), nasofrontal angle (soft tissue glabella-soft tissue nasion-pronasale), nasal tip projection (perpendicular distance between soft tissue nasion to pronasale), nasal length (soft tissue nasion to pronasale), alar base width (alar curvature to alar curvature), and nasal width (alare to alare)  B) CBCT (T0 and T12) | 1. Nasolabial angle:  No significant difference between the two groups at T12 (P = 0.98); changes in nasolabial angle:  - Group 1: 1.55° ± 8.53°  - Group 2: 0.84° ± 3.72°  2. Nasal tip angle:  No significant difference between the two groups at T12 (P = 0.19); changes in nasal tip angle:  - Group 1: 1.25° ± 3.09°  - Group 2: –0.08° ± 2.00°  3. Nasofrontal angle:  Significant difference between the two groups at T12 (P < 0.05); –1.35° ± 2.89° (Group 1) >> –0.10° ± 2.03° (Group 2)  4. Nasal tip projection:  No significant difference between the two groups at T12 (P = 0.99)  5. Nasal length:  Significant difference between the two groups at T12 (P < 0.05); –0.62 ± 0.78 mm (Group 1) >> –0.07 ± 0.42 mm (Group 2)  6. Nasal width:  Significant difference between the two groups at T12 (P < 0.01); 1.46 ± 1.08 mm (Group 1) >> –0.81 ± 0.64 mm (Group 2)  7. Alar base width:  Significant difference between the two groups at T12 regarding the amount of increase in alar base width (P < 0.01); 1.99 ± 1.40 mm (Group 1) >> 0.54 ± 0.67 mm (Group 2)  **** Conclusions:***  Sub-spinal LF-IO with reduction of the piriform aperture, alar base cinch suture, and V-Y closure can prevent undesirable transverse soft tissue changes of the nose. |
| A) Taxis et al. (2024) (32)  B) Non-randomized clinical trial | A) 102  B) 42:60  C) Mean: 28.12 ± 9.41 / Range: 17-56 | Maxillary movements:  - Cranial (n = 36)  - Anterior (n = 96)  - Caudal (n = 18)  **** Note:***  1. Nasal septum was released in all cases  2. The nasal septum was refixed centrally via a drill hole on the anterior  nasal spine in all cases | A) Class II (n = 50) and class III (n = 47) malocclusions, circular open bite (n = 2), anterior open bite (n = 1), mandibular retrognathism (n = 1), and mandibular prognathism (n = 1)  B) Previous orthognathic surgeries; cleft jaw and palate; presence of syndromes | A) Single-piece LF-IO (n = 102)  B) Cartilaginous nasal septum was shortened and straightened  C) Nasotracheal intubation  D) 4 L-shaped titanium plates | A) Nasal septum angle and deviation  B) CBCT (T0 and T6) | 1. Nasal septum angle:  1.1. An increase of 0° to 0.5° in 33 cases (32.3%)  1.2. An increase of 0.5° to 3.0° in 27 cases (26.5%)  1.3. An increase of 3.0° to 6.0° in 2 cases (2%)  1.4. A decrease of 0° to 0.5° in 18 cases (17.6%)  1.5. A decrease of 0.5° to 3.0° in 15 cases (14.7%)  1.6. A decrease of 3.0° to 6.0° in 2 cases (2%)  1.7. No change in nasal septum angle was observed in 5 cases (4.9%)  2. Nasal septum deviation:  2.1. A mean of 5.28° ± 2.12° deviation was seen in the 62 cases that had an increase in their nasal septum angle (P < 0.001)  2.2. A mean decrease of 4.47° ± 2.36° was seen in the 35 cases that had a decrease in their nasal septum angle (P < 0.001)  3. There was a meaningful positive correlation between patients’ age and the increase in nasal septum angle (r = 0.28, P = 0.014, CI: –1.0 - –0.068)  4. There was a meaningful positive correlation between the maxillary anterior displacement and the decrease in nasal septum angle (r = 0.269, P = 0.042, CI: 0.006-1.0)  **** Conclusions:***  The risk of increasing deviation after LF-IO rises with age. An appropriate ostectomy of the posterior bony septum reduces the risk of septal deviation after maxillary advancement. |
